# Single-cell RNA sequencing for the identification of early-stage lung cancer biomarkers from circulating blood

**DOI:** 10.1038/s41525-021-00248-y

**Published:** 2021-10-15

**Authors:** Jinhong Kim, Zhaolin Xu, Paola A. Marignani

**Affiliations:** 1 Department of Biochemistry and Molecular Biology, Faculty of Medicine, Dalhousie University, Room 9F1, 5850 College Street, Halifax, Nova Scotia B3H1X5 Canada; 2 Department of Pathology, Faculty of Medicine, Dalhousie University, Room 734C, 5788 University Avenue, Halifax, Nova Scotia B3H1V8 Canada

**Keywords:** Next-generation sequencing, Non-small-cell lung cancer

## Abstract

Lung cancer accounts for more than half of the new cancers diagnosed world-wide with poor survival rates. Despite the development of chemical, radiological, and immunotherapies, many patients do not benefit from these therapies, as recurrence is common. We performed single-cell RNA-sequencing (scRNA-seq) analysis using Fluidigm C1 systems to characterize human lung cancer transcriptomes at single-cell resolution. Validation of scRNA-seq differentially expressed genes (DEGs) through quantitative real time-polymerase chain reaction (qRT-PCR) found a positive correlation in fold-change values between C-X-C motif chemokine ligand 1 (*CXCL1*) and 2 (*CXCL2*) compared with bulk-cell level in 34 primary lung adenocarcinomas (LUADs) from Stage I patients. Furthermore, we discovered an inverse correlation between chemokine mRNAs, miR-532-5p, and miR-1266-3p in early-stage primary LUADs. Specially, miR-532-5p was quantifiable in plasma from the corresponding LUADs. Collectively, we identified markers of early-stage lung cancer that were validated in primary lung tumors and circulating blood.

## Introduction

Globally, 18 million people were diagnosed with various cancers in 2018. The mortality rate for patients with lung cancer was twice (18.4%) that of other cancers, including colorectal (9.2%), breast or stomach (6.6%), and liver (8.2%) cancers^1^. Lung cancer represents more than half of all new cancers diagnosed in North America^2,3^ and is predominantly associated with smoking behavior. The 5-year-survival rate for lung cancer patients diagnosed at stage IV is significantly poorer (19%) compared with 55% for stage I diagnosis, as well as that of prostate, breast, and colon cancer patients of which the 5-year-survival rates are 95%, 88%, and 64%, respectively^2^. For non-small cell lung carcinoma (NSCLC), the most common form of lung cancer, molecular diagnostic technologies are based on a small number of biomarkers using a curated panel of oncogenes^4^ that form the basis for targeted therapies. Common genomic alterations occur in *EGFR, HER2, KRAS, c-MYC*, and *ALK* genes. Therapeutic targeting of altered genes has modestly improved clinical outcomes, for example, ~20–30% of NSCLC express enhanced levels of the mutated *EGFR*, warranting treatment with the inhibitor Iressa that permits progression-free survival for up to 10 months^5^ with up to 60% of patient developing resistance to treatment or acquiring additional mutations^6^. Therefore, it is paramount that novel molecular biomarkers for diagnosis of lung cancers at early stage are discovered in order to improve survivorship and quality of life through early detection.

Cancers are comprised of tumor cell populations with diverse transcriptional programs that contribute to the complexity of the cancer and are considered primary contributors to therapy resistance, recurrence, and poor prognosis^7^. Recent innovations in next-generation sequencing (NGS) and microfluidics technologies are enabling scientists to profile differential gene expressions at the level of single cells using scRNA-seq applications. In comparison with conventional RNA-seq (bulk RNA-seq), innovative scRNA-seq applications facilitate the detection of DEGs within individual cells and across cell populations. The new knowledge gained from scRNA-seq analysis will contribute to the identification of predictive biomarkers that will lead to improvements in molecular diagnostic screening panels and discovery of personalized cancer therapies that take into consideration the uniqueness of an individual cancer.

For this study, we performed a 3′-end scRNA-seq analysis using Fluidigm C1 systems to identify new candidate genes that could serve as molecular biomarkers for diagnosis of lung cancers. We used four human NSCLC epithelial cell lines, A549, H460, H1299, and Calu3, for gene expression profiling at single-cell resolution followed by validation in primary lung tumors against tumor-adjacent normal lung tissues (hereafter normal lung tissues) resected from 34 early-stage LUAD patients. We discovered differential expression of microRNAs that regulate chemokine mRNA expressions through epigenetic means by further validating blood (plasma) samples collected from corresponding LUAD patients at the time of surgical resection. Overall, the detection of DEGs at single-cell resolution followed by successful validation in lung cancer cells, primary tumors, and circulating blood has enabled the identification of novel molecular markers that can be used for diagnosis of early-stage lung cancer.

## Results

### Construction of dual-indexed and 3′-end enriched cDNA libraries for scRNA-seq

**Fig. 1:**
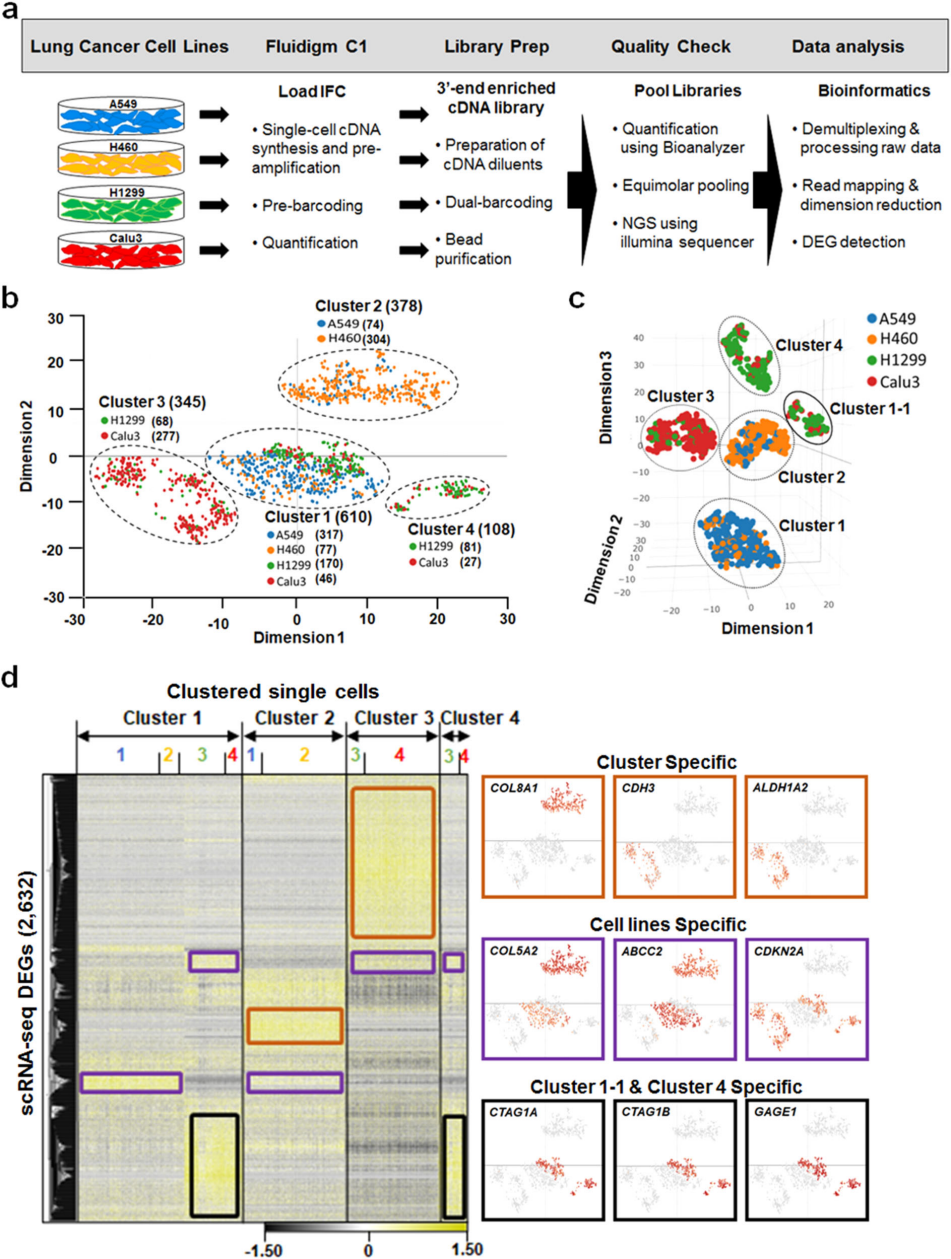
Single-cell RNA-seq workflow and clustering analyses. **a** Individual cells from A549 (blue), H460 (orange), H1299 (green), and Calu3 (red) were captured in separate Fluidigm C1 HT IFCs and pre-indexed with Fluidigm cell-specific barcodes at 3′-end of polyadenylated mRNAs during pre-amplification of cDNAs synthesized from total RNAs isolated from single cells, followed by library construction. Dual-indexed and 3′-end enriched cDNA libraries (*n* = 1,600) were sequenced in Illumina NextSeq 500 systems, followed by DEG detection. **b** Single cells (*n* = 1,441) in clusters (*n* = 4) re-arranged from NSCLC cell lines (*n* = 4). Parentheses include the number of cells in clusters or cell lines. **c** Cluster presentation in three dimensions. Subcluster, Cluster 1–1, is presented between Cluster 2 and 4. **d** Heatmap analysis of DEGs (*n* = 2,632). Z-scores of the read-count DEG dataset were adjusted from −1.50 (black) to 1.50 (yellow). Color-matching numbers represent A549 (blue; 1), H460 (orange; 2), H1299 (green; 3), and Calu3 (red; 4) as shown in (**a**–**c**). DEGs specific to a cluster or cell line are highlighted in orange and purple, respectively. DEGs specific to Cluster 1–1 and Cluster 4 are highlighted in black. Representative DEGs per cluster, cell line and, Cluster 1–1 & Cluster 4-specific expression shown in the color-matching panels. See Supplementary Data 1 for full gene names. All DEGs are statistically significant at FDR-corrected *P* value < 0.05 showing fold changes >|2|.

The transcriptomes of human NSCLC were characterized at single-cell resolution through profiling DEGs. Mature mRNA molecules were isolated from 1,600 individual cells of four NSCLC epithelial cell lines (400 cells per cell line), A549, H460, H1299, and Calu3 (Fig. 1a), commonly used for lung cancer studies. We applied a dual-barcoding approach for indexing NGS read sets generated from cDNA libraries of individual single cells. First, the mRNA molecules isolated from individual cells were pre-indexed with Fluidigm cell-specific barcodes at 3′-end regions behind poly-A tails during synthesis and pre-amplification of cDNAs in the Fluidigm C1 systems. For second indexing, Illumina Nextera barcode-containing primers were annealed to 5′-end regions of fragmented cDNAs during 3′-end enrichment following tagmentation of pre-indexed individual cDNAs. From sequencing and demultiplexing a total of 1,600 individually dual-indexed and 3′-end enriched cDNA libraries, we obtained ~180 million (M) raw sequence reads (Supplementary Table 1). Following read mapping of processed read sets to human genome (GRCh38.p13; NIH), an average of 5,937 genes per cell line was aligned with reads per gene ≥4 in individual cells (Supplementary Fig. 1a). A total of 24,424 genes were mapped with high-quality reads per cell >2,000 (Supplementary Fig. 1b), and raw read counts were normalized at count per million (CPM) reads per gene ≥1 (Supplementary Fig. 1b).

### Detection of DEGs

**Fig. 2:**
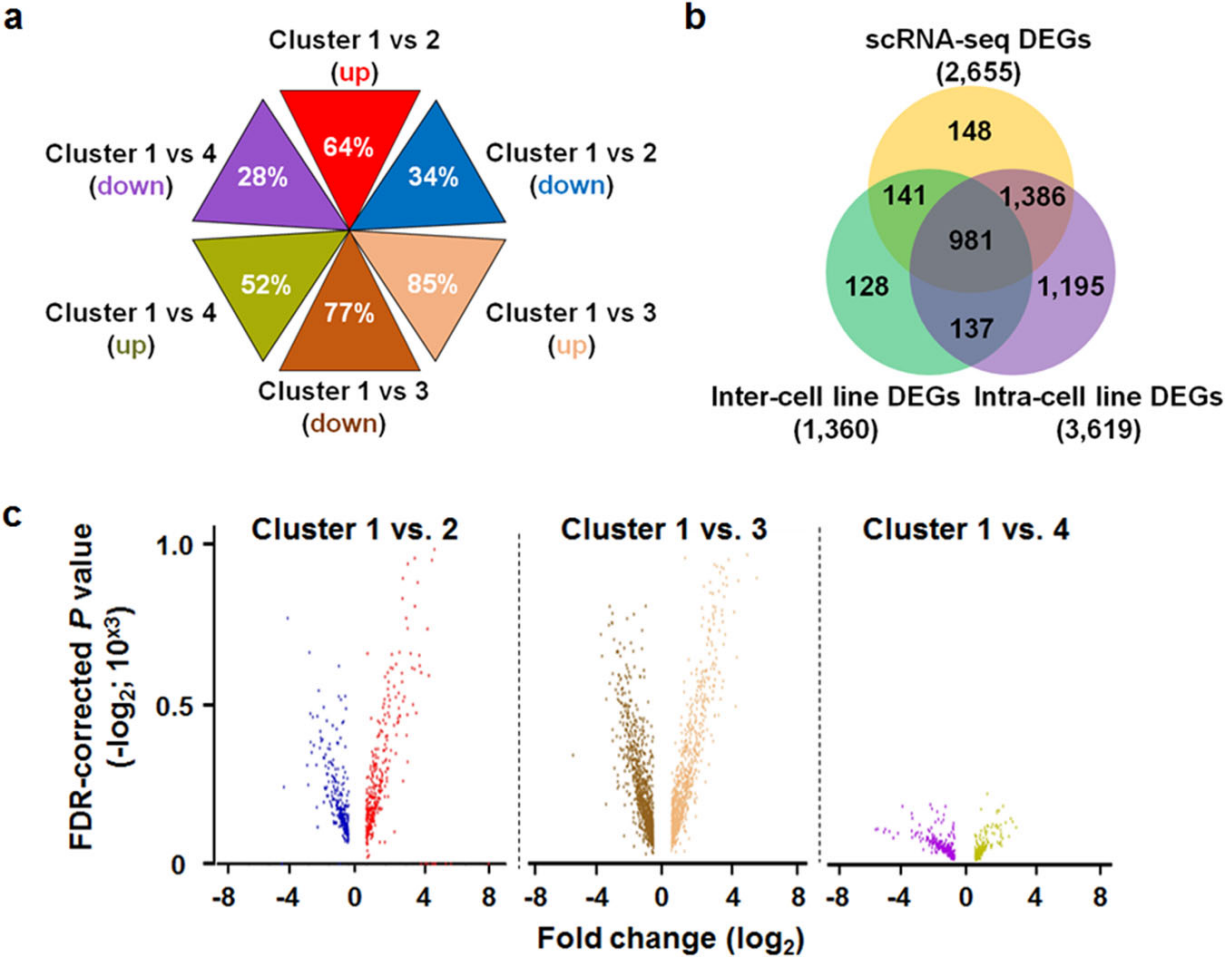
Characterization of DEGs detected from Fluidigm 3′-end scRNA-seq dataset. **a** Hexagonal triangle diagram indicates the percentage of scRNA-seq DEGs uniquely detected in up- or down-regulated gene set per cluster comparison. **b** Venn diagram presenting scRNA-seq DEGs (*n* = 2,655), intra-cell line DEGs (*n* = 1,360), and inter-cell line DEGs (*n* = 3619). Individual values indicate the number of unique or overlapping DEGs among the three DEG sets. **c** Volcano plots showing correlation between values of scRNA-seq DEG fold change (log_2_; *x* axis) and FDR-corrected *P* (<0.05; −10^3^ × log_2_; *y* axis) per up- or down-regulated gene set from cluster comparison. Individual DEGs are presented in the color-matching dots per gene set as in (**a**).

We performed a t-distributed stochastic neighbor embedding (t-SNE) analysis to reduce dimensionality of normalized gene expression values that are highly variable dataset. Then, a complete-linkage hierarchical clustering analysis was conducted using an unsupervised single-cell consensus clustering tool, SC3 (RRID:SCR_015953), and re-arranged single cells from the four human NSCLC cell lines into four different clusters based on differential gene expression in two dimensions (Fig. 1b). Interestingly, each cluster contained cells from two cell lines, and cells per cluster were re-arranged from one of two cell lines (Fig. 1b and Supplementary Table 2). Although Cluster 1 was comprised of cells from all four NSCLC cell lines (Fig. 1b and Supplementary Table 2), A549 cells represented 52% (317/610) of cells re-arranged into Cluster 1. Three-dimensional representation of the four clusters identified a subcluster, Cluster 1–1 (Fig. 1c), within Cluster 1 comprised of 170 and 46 cells from H1299 and Calu3, respectively. To minimize complexity when comparing clusters for DEG detection, we employed the clustering result in two dimensions. Cluster 1 was positioned in the center of cluster plot where perplexity values determining the extent of cluster density were closed to ‘0’ in two dimensions (Fig. 1b). We set Cluster 1 as the control cluster for detection of DEGs to compare with the three other clusters. In total, 2,655 DEGs were detected from three comparisons; Cluster 1 vs. Cluster 2, Cluster 1 vs. Cluster 3, and Cluster 1 vs. Cluster 4 (Supplementary Data 1). DEGs were identified by fold-change differences ≥|2| in normalized expression values per gene at the statistical significance of false discovery rate (FDR)-corrected *P* value < 0.05. Following cross check of gene ID between Ensembl and NCBI gene databases, we categorized 2,632 scRNA-seq DEGs into cluster-specific or cell-line-specific DEGs using a heatmap-clustering analysis (Fig. 1d). Hierarchical sorting of heatmap-clustered DEGs according to expression patterns clearly distinguished cluster-specific DEGs (highlighted in orange), and cell line-specific DEGs (highlighted in purple) (Fig. 1d). A third DEG set (highlighted in black) was identified in H1299 and Calu3 cells that were re-arranged into Cluster 1–1 (Fig. 1c) and Cluster 4 (Fig. 1d). Furthermore, when all 2,655 DEGs were divided into six different gene sets based on up- or down-regulation, we detected DEGs unique to single gene sets (Fig. 2a). For example, 85% (beige triangle, 802/939; Supplementary Data 1) of genes up-regulated in Cluster 3 compared with Cluster 1 were uniquely detected from only the cluster comparison, and 28% (63/226; Supplementary Data 1) of genes down-regulated in Cluster 4 compared with Cluster 1 (purple triangle in Fig. 2a) did not overlap scRNA-seq DEGs detected in other cluster comparisons. To further characterize the scRNA-seq DEGs, we prepared two additional DEG sets as following: (1) inter-cell line DEGs were detected by individually comparing normalized gene expression values in cells from A549 with those from H460, H1299, and Calu3; and (2) intra-cell line DEGs were prepared by individually comparing normalized gene expression values in cells from a cell line in a cluster with those in cells from an identical cell line but existing in different clusters. For example, to detect intra-cell line DEGs for A549, normalized gene expression values in A549 cells in Cluster 1 were compared with those in A549 cells in Cluster 2. When comparing the DEG sets, we identified a significantly large portion (90%; 2,367 of 2,655) of scRNA-seq DEGs overlapped with a set of intra-cell line DEGs (Fig. 2b). Moreover, to determine the extent of statistical significance in detecting scRNA-seq DEGs, we generated three volcano plots using two values of FDR-corrected *P* (−10^3^ × log_2_) and fold change (log_2_) per up- or down-regulated gene in the comparisons of Cluster 1 with Cluster 2 and Cluster 3 (Fig. 2c). Those plots presented overall positive correlations, that is, the higher absolute values of fold change of scRNA-seq DEGs were detected at the more statistically significant level. However, the volcano plot from Cluster 1 vs. Cluster 4 revealed relatively lower correlation between the two values when compared with other two volcano plots (Fig. 2c) due to the similarity in gene expression pattern between Cluster 1–1 and Cluster 4 (highlighted in black; Fig. 1d) that offset the extent of differential gene expression.

### Gene set enrichment analysis (GSEA)

**Fig. 3:**
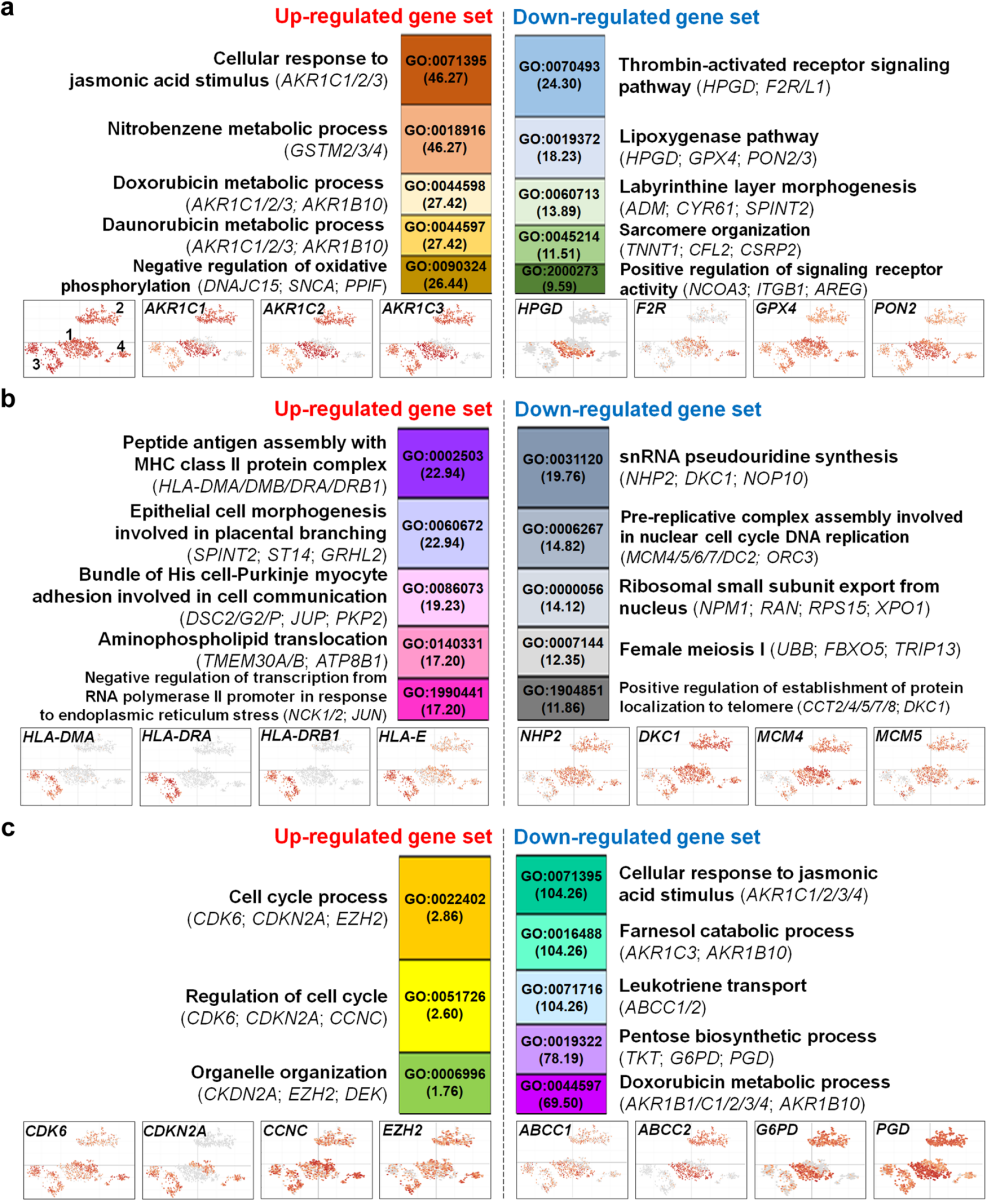
GO of biological process overrepresented from GSEA. **a**–**c** Top 5 GO terms enriched with up (*n* = 355)- and down (*n* = 305)-regulated genes in Cluster 2 in (**a**), up (*n* = 939)- and down (*n* = 1,172)-regulated genes in Cluster 3 in (**b**) and up (*n* = 199)- and down (*n* = 226)-regulated genes in Cluster 4 in (**c**) when individually compared with Cluster 1 (Control cluster). The numbers in parentheses below GO IDs in individual bars and gene names in parentheses below GO descriptions indicate enrichment values at FDR-corrected *P* value < 0.05 and primary contributors to overrepresentation of the top 5 biological processes per gene set, respectively. Expression maps are presented below GO bars to visualize differential expression of selected DEGs. Reference expression map shown in lower panel (**a**) contains information on a position of clusters in two dimensions. See Supplementary Data 1 for full gene names.

To identify functional annotations enriched in up- or down-regulated gene set per cluster comparison, we used six DEG sets comprised of up- or down-regulated genes in Cluster 2, Cluster 3, and Cluster 4 against Cluster 1 allowing to include DEGs commonly detected in more than two cluster comparisons (Supplementary Data 1). We conducted enrichment analyses with the six DEG sets using three biological databases, the Gene Ontology (GO), KEGG pathway, and Molecular Signatures. The Molecular Signatures Database is comprised of gene sets >30,000 in nine different collections that were registered from various research projects. For GSEA in the current study, we used the collection 6 (C6) comprised of 189 oncogenic signature gene sets that were primarily identified by a microarray analysis of various cancers. For a full list of overrepresented GO terms, KEGG pathways, and oncogenic gene sets, see Supplementary Data 2, 3, and 4, respectively. Briefly, a GSEA using the GO database resulted in overrepresentation of 441 unique GO terms (Supplementary Table 3) which were hierarchically located at the lowest position (GO level is ‘0’), indicating the most specific functional annotations in the GO category of biological processes. Overall, there were some specific genes or gene families, such as glutathione S-transferase mu 2/3/4 (*GSTM2/3/4*) (Fig. 3a), aldo/keto reductase gene family (Fig. 3a and c), HLA class II histocompatibility antigen gene family (Fig. 3b), cell cycle-associated genes (cyclin-dependent kinases and cyclin-dependent kinase inhibitors) (Fig. 3c) or ATP binding cassette subfamily C member 1/2 (Fig. 3c), that contributed to significant overrepresentation of biological processes ranked as top 5 GO terms per up- or down-regulated gene set. Furthermore, we identified a total of 79 unique KEGG pathways enriched with the six DEG sets from a second GSEA (Supplementary Table 3). We found that some specific genes or gene families commonly contributed to significant overrepresentation of GO terms and KEGG pathways. For example, glutathione metabolism (KEGG pathway ID: hsa00480) in Cluster 1 vs. Cluster 2, all top 5 pathways in Cluster 1 vs. Cluster 3 and steroid hormone biosynthesis (hsa00140) in Cluster 1 vs. Cluster 4, were enriched by *GSTM2/3/4* gene family, HLA class I/II histocompatibility gene families and aldo/keto reductase gene family, respectively (Table 1). Moreover, we found a total of 72 unique oncogenic gene sets overrepresented from a third GSEA using the Molecular Signatures database (Supplementary Table 3). In particular, two oncogenic gene sets, CORDENONSI_YAP_CONSERVED_SIGNATURE (Oncogenic gene set ID: M2871) and SINGH_KRAS_DEPENDENCY_SIGNATURE (M2851), were comprised of relatively more reference genes overlapping with our scRNA-seq DEGs (42%; M2851 and 40%; M2851, respectively) when compared with other overrepresented oncogenic gene sets (Supplementary Data 4). In addition, the SINGH_KRAS_DEPENDENCY_SIGNATURE is comprised of 20 reference genes associated with cell viability and positively correlated with *KRAS* mutation for stable gene expression^8^. Eight of the 20 reference genes overlapped with our up-regulated genes in Cluster 3 against Cluster 1, and the dominant cell lines of the Cluster 1 and Cluster 3 were *KRAS*-mutated A549 and wild-type *KRAS* Calu3, respectively^9^ (Supplementary Data 4). Four of the 8 overlapping reference genes were chromosome 1 open reading frame 116 (*C1orf116*)^10^, laminin subunit (*LAM*) gene family^11^, ladinin 1 (*LAD1*)^12^, and amphiregulin (*AREG*)^13^, which are associated with regulatory process in epithelial-mesenchymal transition (EMT). However, oncogenic gene sets resulting from differential expression and/or mutations on *KRAS* or *TP53* that are the most frequent in lung cancer cells^9^ were not constitutively enriched with our six DEG sets (Supplementary Data 4).

**Table 1:** Top five KEGG pathways and molecular signatures overrepresented from GSEA.

Database	Biological process ID	ID description	Gene name^a^	Regulation (Cluster ID^b^)	EV^c^	FDR-corrected *P* value
KEGG pathways	hsa00480	Glutathione metabolism	*GSTM2/3/4, GPX1/3, GSTO2*	Up (C2)	8.86	*1.16E−03*
	hsa05134	Legionellosis	*CXCL1/2/8, IL6, CASP7/8*		7.34	*2.15E−03*
	hsa00980	Metabolism of xenobiotics by cytochrome P450	*GSTM2/3/4, GSTO2, AKR1C1*		5.37	*3.94E−02*
	hsa05146	Amoebiasis	*CXCL1/2/8, IL6, COL4A6*		4.15	*3.94E−02*
	hsa04621	NOD-like receptor signaling pathway	*CXCL1/2/8, IL6, NAMPT*		3.51	*2.40E−02*
	NA	NA	NA	Down (C2)	NA	NA
	hsa05330	Allograft rejection	*HLA-A/B/C/E, HLA-DMA/B, HLA-DPA1/B1*	Up (C3)	16.60	*4.80E−07*
	hsa05320	Autoimmune thyroid disease			16.60	*4.80E−07*
	hsa05332	Graft-versus-host disease			13.58	*1.44E−06*
	hsa04940	Type I diabetes mellitus			10.80	*1.77E−06*
	hsa04612	Antigen processing and presentation			5.95	*3.46E−06*
	hsa03050	Proteasome	*PSMA3/4, PSMB1/5/6/7, PSMC2/3/4/5/6*	Down (C3)	9.10	*8.28E−09*
	hsa03030	DNA replication	*MCM4/5/6/7, RFC2/3, RPA1/2*		8.50	*2.69E−07*
	hsa00190	Oxidative phosphorylation	*COX7A2L8A/5A, NDUFA1/4/6/8/9, NDUFB1/2/4/6/7/8*		7.76	*2.01E−10*
	hsa05012	Parkinson’s disease	*COX5B/6B1/6C, NDUFA1/4/6/8/9, NDUFB1/2/4/6/7/8*		7.28	*0*
	hsa03010	Ribosome	*MRPL1/12/13/15, MRPS2/7/10/20, RPL6/7/7A/18A*		6.86	*1.24E−10*
	NA	NA	*NA*	Up (C4)	NA	*NA*
	hsa00030	Pentose phosphate pathway	*G6PD, PGD, PFKP*	Down (C4)	10.99	*1.48E−02*
	hsa00480	Glutathione metabolism	*GPX1/2, IDH1, MGST1*		9.55	*7.37E−03*
	hsa00590	Arachidonic acid metabolism	*GPX1/2, AKR1C3, LTA4H*		8.59	*1.48E−02*
	hsa00140	Steroid hormone biosynthesis	*AKR1C1/2/3/4, CYP1B1*		8.05	*4.16E−02*
	hsa00980	Metabolism of xenobiotics by cytochrome P450	*AKR1C1, ALDH3A1/B1 CYP1B1*		7.98	*1.01E−02*
Molecular Signatures	M2697	P53_DN.V1_DN	*CXCL1, EPB41L3, CPS1*	Up (C2)	4.58	*1.67E−05*
	M2725	MEK_UP.V1_UP	*GAL, COL5A2, LIMCH1*		4.35	*1.67E−05*
	M2892	KRAS.KIDNEY_UP.V1_UP	*EPB41L3, LIMCH1, NEFL*		3.82	*1.09E−02*
	M2900	KRAS.LUNG.BREAST_UP.V1_UP	*CXCL1/2/5/8, RPS4Y1, GLRX*		3.79	*1.58E−02*
	M2634	EGFR_UP.V1_UP	*GAL, COL5A2, SCCPDH*		3.46	*2.16E−03*
	M2871	CORDENONSI_YAP_CONSERVED_SIGNATURE	*AXL, SERPINE1, MARCKS*	Down (C2)	7.25	*1.37E−03*
	M2634	EGFR_UP.V1_UP	*KRT81, AKAP12, KRT7*		5.14	*1.12E−05*
	M2698	P53_DN.V1_UP	*AXL, SPINT2, SCRN1*		5.11	*1.12E−05*
	M2725	MEK_UP.V1_UP	*KRT7/81, COTL1, ARL4C*		5.02	*1.12E−05*
	M2769	ESC_V6.5_UP_EARLY.V1_DN	*AXL, SERPINE1, GPX2*		4.56	*9.38E−04*
	M2851	SINGH_KRAS_DEPENDENCY_SIGNATURE	*C1orf116, LAMC2, LAD1*	Up (C3)	7.60	*1.14E−03*
	M2768	ESC_J1_UP_LATE.V1_UP	*CTGF, SPINK1, CTSH*		4.28	*0*
	M2790	EIF4E_DN	*C3, FNDC3B, NEDD4L*		4.25	*1.02E−06*
	M2698	P53_DN.V1_UP	*CD24, KRT19, EPCAM*		4.04	*0*
	M2903	LEF1_UP.V1_DN	*CFTR, PDZK1IP1, CXCL5*		3.81	*2.78E−09*
	M2660	CSR_LATE_UP.V1_UP	*MT2A, DTYMK, GTF3C6*	Down (C3)	7.22	*0*
	M2871	CORDENONSI_YAP_CONSERVED_SIGNATURE	*SERPINE1, BIRC5, GGH*		4.68	*2.73E−04*
	M2800	RB_DN.V1_UP	*PCNA, RAD51C, MCM7*		4.43	*9.14E−08*
	M2791	EIF4E_UP	*ATP5MF, MDH2, NOP16*		4.26	*5.97E−05*
	M2675	VEGF_A_UP.V1_DN	*CKS2, CCNB1, MCM4*		4.23	*5.17E−10*
	M2660	CSR_LATE_UP.V1_UP	*MT2A, BEX1, EZH2*	Up (C4)	5.63	*2.54E−03*
	M2905	LEF1_UP.V1_UP	*CDKN2A, G0S2, FHL1*		4.60	*9.61E−03*
	M2698	P53_DN.V1_UP	*CDKN2A, G0S2, SOX9*		4.55	*7.70E−03*
	M2725	MEK_UP.V1_UP	*KRT81, S100A6, AKR1C2*	Down (C4)	7.74	*5.09E−10*
	M2780	NFE2L2.V2	*AKR1C1/3, AKR1B10, MGST1*		7.05	*5.09E−10*
	M2634	EGFR_UP.V1_UP	*KRT7/81, PCDH9, EDN1*		5.75	*3.03E−06*
	M2636	ERBB2_UP.V1_UP	*KRT81, S100A6, AKR1C2*		5.75	*3.03E−06*
	M2807	CAHOY_ASTROGLIAL	*AKR1B10, ANXA1, EREG*		5.53	*5.01E−03*

^a^Three representative genes or gene families per overrepresented signaling pathway or oncogenic gene set. Please see the Supplementary Table S3 for full name of genes.

^b^C2-Cluster 2, C3-Cluster 3, and C4-Cluster 4 against Cluster 1.

^c^Enrichment value.

### Validation of selected DEGs using qRT-PCR analysis

**Fig. 4:**
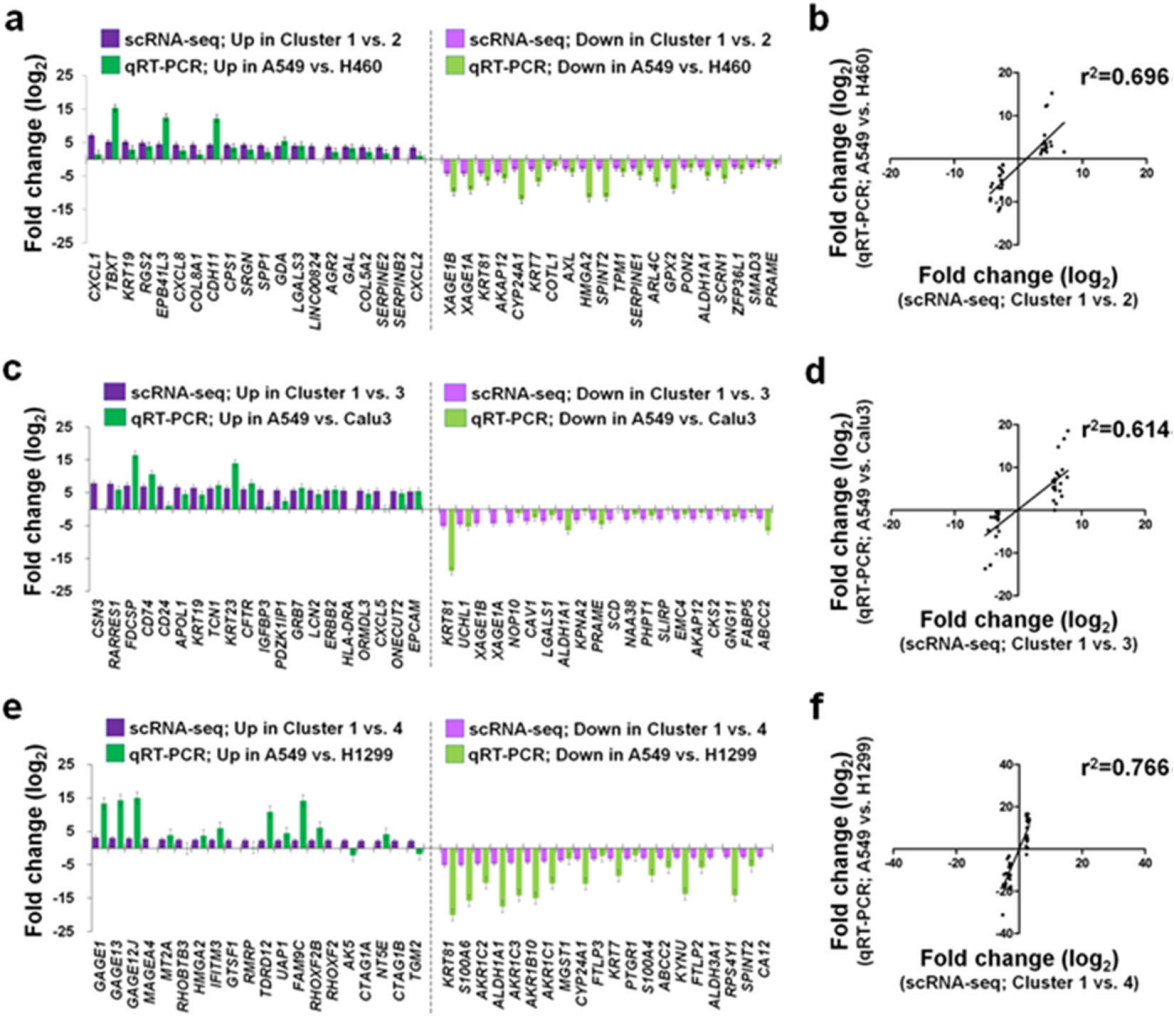
Validation of scRNA-seq DEGs by qRT-PCR analysis. **a**, **c**, **e** Direct comparison of fold change for selected scRNA-seq DEGs (*n* = 120) detected from three cluster comparisons, Cluster 1 vs. Cluster 2 in (**a**), Cluster 1 vs. Cluster 3 in (**c**), and Cluster 1 vs. Cluster 4 in (**e**), compared with genes identified in A549 vs. H460 in (**a**), A549 vs. Calu3 in (**c**), and A549 vs. H1299 in (**e**), using qRT-PCR. Prioritized DEGs (*n* = 120) are comprised of the first 20 up- and the first 20 down-regulated genes (40 DEGs) per cluster comparison. *X* and *y* axes indicate gene names and fold-change values (log_2_), respectively. Fold-change values expressed as mean ± SEM; three separate experiments conducted in duplicate. **b**, **d**, **f** Linear regression analysis was conducted for fold-change values of prioritized 120 selected DEGs between Fluidigm 3′-end scRNA-seq (*x* axes) and qRT-PCR (*y* axes) analyses corresponding to the direct fold-change comparisons in (**a**), (**c**), and (**e**), respectively. Coefficient of determination (*r*^2^) values are presented at *P* value < 0.0001.

We validated fold-change values of DEGs obtained from our scRNA-seq dataset with those from qRT-PCR analysis at bulk-cell level. For this validation, 2,655 scRNA-seq DEGs were ranked from highest to lowest fold-change value (Supplementary Data 1), following this we selected 40 DEGs per cluster comparison (first 20 up-regulated and first 20 down-regulated genes; 120 DEGs in total). We used Cluster 1 as the control cluster for DEG detection (Supplementary Data 1). Because the A549 was the predominant cell line identified in Cluster 1 (Fig. 1b and Supplementary Table 2), we used A549 as the control cell line to obtain fold-change values of the 120 selected DEGs. We then directly compared the fold-change values from the scRNA-seq and qRT-PCR analyses (Fig. 4a, c and e). Overall, the direct comparisons of fold-change values showed positive correlation between the two analyses as indicated in the range of coefficient of determination (*r*^2^) values from 0.61 to 0.77 (Fig. 4b, d and f). Of the 120 selected DEGs, there were only two exceptions, adenylate kinase 5 (*AK5*) and transglutaminase 2 (*TGM2*), presenting an inverse expression that was up-regulated in Cluster 4 against Cluster 1 from our scRNA-seq analysis but down-regulated in H1299 against A549 from the qRT-PCR analysis (Fig. 4e).

### Absolute quantification of chemokine genes and microRNA copy numbers in early-stage LUAD patients

**Fig. 5:**
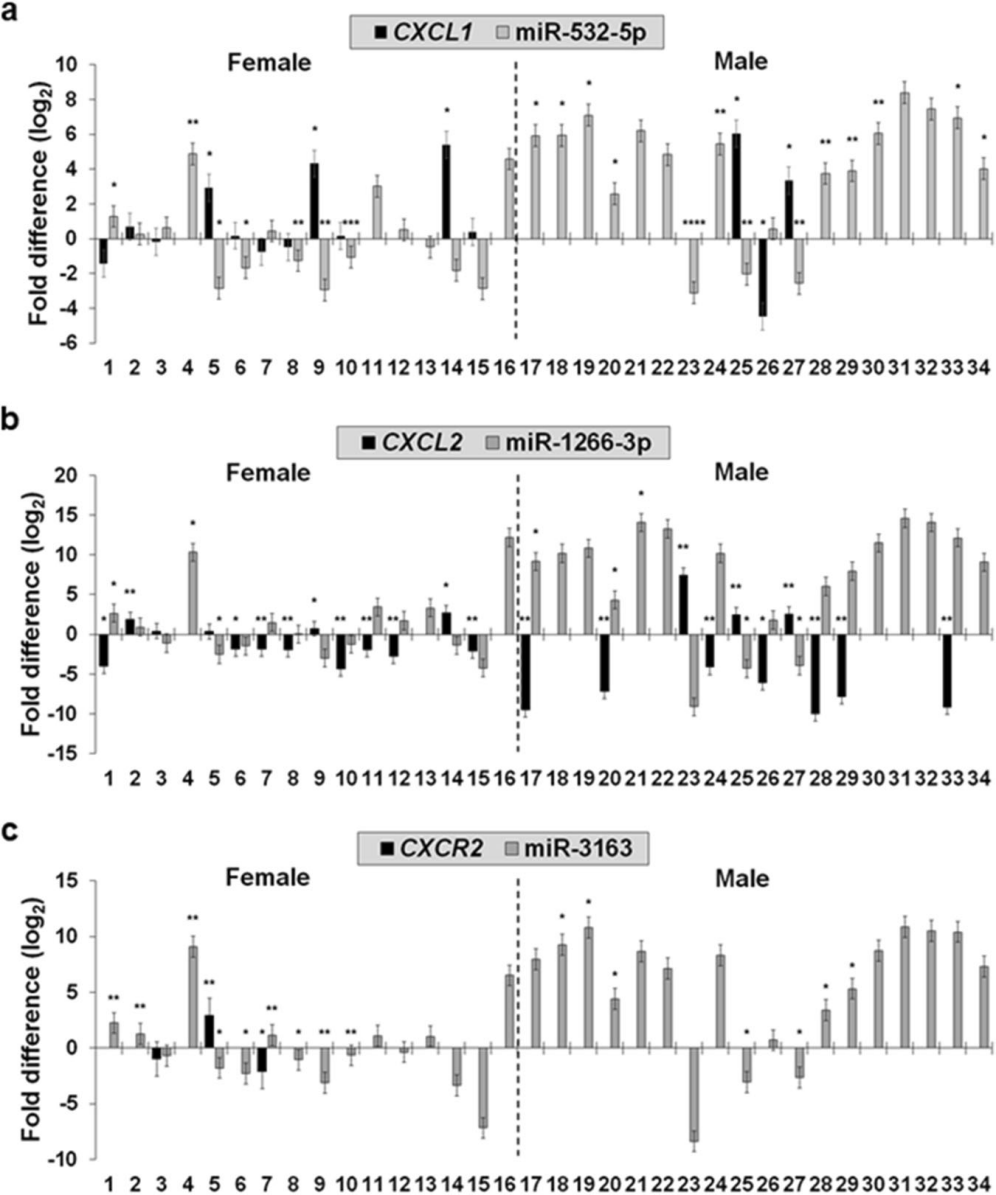
Quantitative analysis of *CXCL* mRNAs and microRNA copy numbers from Stage I LUAD patients. **a**–**c** Absolute quantification of *CXCL1* and miR-532-5p in (**a**), *CXCL2* and miR-1266-3p in (**b**), and *CXCR2* and miR-3163 in (**c**). Black and gray bars present quantitative fold differences of chemokine genes (*n* = 3) and corresponding miRNAs (*n* = 3), respectively, in primary lung tumors compared with normal lung tissues resected from female (*n* = 16) and male (*n* = 18) Stage I LUAD patients. *x* and *y* axes indicate patient IDs (female patients, 1–16 and male patients, 17–34) and quantitative fold differences at log_2_, respectively. Statistical differences indicated as *P < 0.05, **P < 0.01, ***P < 0.001, and ****P < 0.0001 from two-sample *t*-tests.

In NSCLC, tumor-associated antigens (TAAs) are aberrantly expressed^14^. In our scRNA-seq DEG dataset, 13 TAAs from 6 different gene families were found in the 20 first up-regulated and 20 first down-regulated genes from all three cluster comparisons (Fig. 4a, c and e). Using a qRT-PCR analysis, we pre-screened the extent (cycle threshold; Ct) of those TAA expressions in primary lung tumors resected from 34 (16 female and 18 male) Stage I LUAD patients (Supplementary Data 5). Notably, all four tested *CXCL* gene family (*CXCL1*, *CXCL2*, *CXCL5*, and *CXCL8*) showed higher successful amplification rates in primary lung tumors when compared with other TAAs (Supplementary Data 5). Thus, we validated *CXCL* gene family members, specifically *CXCL1* and *CXCL2* that were the first up-regulated in Cluster 2 against Cluster 1 (Fig. 4a) and amplified in almost all primary lung tumors (Supplementary Data 5), respectively. For this validation, we employed a standard curve approach for absolute quantification of the two chemokine genes to identify a difference in quantities in 34 early-stage LUAD patient primary lung tumors against patient-matched normal lung tissues. C-X-C motif chemokine receptor 2 (*CXCR2*) was also included for this quantification. The quantities of *CXCL2* (Supplementary Fig. 2b) were a maximum of 104 times and 15 times higher in normal lung tissues and primary lung tumors, respectively, when compared with those of *CXCL1* (Supplementary Fig. 2a). The quantitative difference between the two chemokine genes corresponded to the result from pre-screening Ct values of those genes showing a lower averaged Ct value (31.44) of *CXCL2* compared with that (35.61) of *CXCL1* (Supplementary Data 5). Both *CXCL1* and *CXCL2* were quantifiable in most normal lung tissues (32 and 34 LUAD patients, respectively) (Supplementary Fig. 2a and b, respectively). However, *CXCL1* was not detectable in 5 female and 14 male tumor tissues (19 patients in total) (Supplementary Fig. 2a), and *CXCL2* was not quantifiable in tumor tissues of 3 female and 8 male patients (11 patients in total) (Supplementary Fig. 2b). In addition, we employed an absolute quantification approach to investigation of three human microRNAs, miR-532-5p, miR-1266-3p, and miR-3163, that epigenetically regulate a mRNA expression of *CXCL1*, *CXCL2*, and *CXCR2*, respectively^15^ (Supplementary Fig. 3). Interestingly, the three chemokine mRNAs and the corresponding microRNAs were inversely correlated for most LUAD patients (Fig. 5).

### Absolute quantification of miR-532-5p, miR-1266-3p, and miR-3163 in plasma samples

**Fig. 6:**
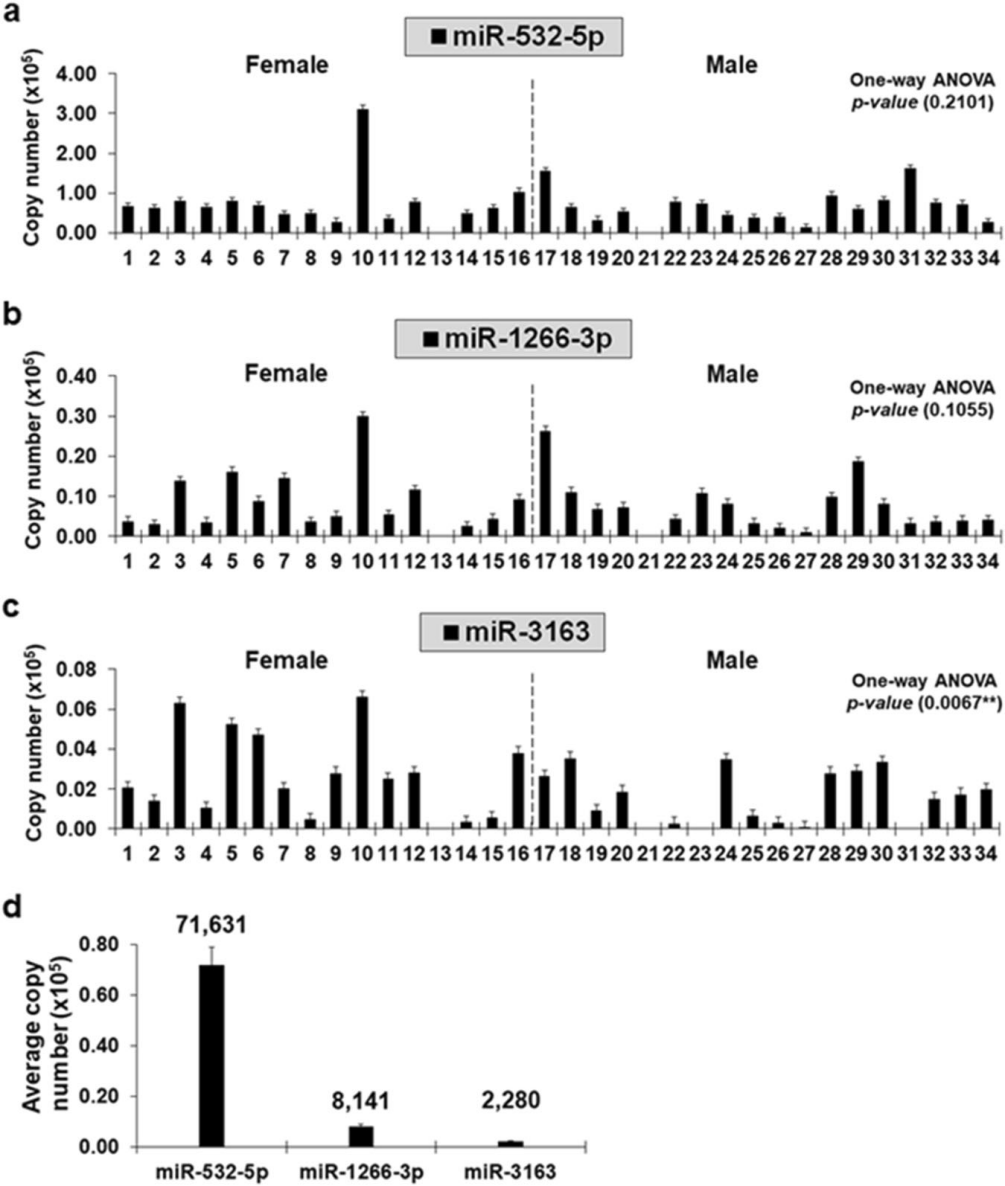
MicroRNA copy numbers found in plasma from Stage I LUAD patients. **a**–**c** Copy numbers of miR-532-5p in (**a**), miR-1266-3p in (**b**), and miR-3163 in (**c**) obtained from absolute quantification using qRT-PCR analysis of plasma isolated at the time of surgical resection from early-stage female (*n* = 16) and male (*n* = 18) LUAD patients. *x* and *y* axes indicate patient IDs (female patients, 1–16, and male patients, 17–34) and measured copy numbers (×10^5^), respectively. **d** Averaged copy number of miR-532-5p, miR-1266-3p, and miR-3163. A standard curve approach was applied to measure copy numbers by qRT-PCR analysis from three separate experiments conducted in duplicate, mean ± SEM; one-way ANOVA analysis, **P < 0.01.

Next, we examined whether miR-532-5p (Fig. 6a), miR-1266-3p (Fig. 6b), and miR-3163 (Fig. 6c) were detectable in blood (plasma) of LUAD patients. Here, we applied an absolute quantification method to obtain copy numbers of miRNAs in plasma of whole blood samples collected from the 34 early-stage LUAD patients at the time of surgical resection. Relative quantification of the miRNAs was not possible because we did not have access to blood from these patients prior to their lung cancer diagnosis. Instead, we used *Caenorhabditis elegans* microRNA 39 (cel-miR-39) miRNA mimic (Qiagen, Inc.) as a normalization control to generate a standard curve in the range of copy numbers from 1 × 10^6^ to 8 × 10^3^ that was sufficient to cover copy numbers of miRNAs of interest in plasma samples. We also used U6 spliceosomal small nuclear RNAs (snRNAs) to obtain a correction factor for normalization of raw Ct values. We found an average of 71,631 copies of miR-532-5p in 33 of 34 plasma samples, while the averaged copy numbers of miR-1266-3p and miR-3163 were relatively less (Fig. 6d).

### Validation of differential expression of *CXCL1*, *CXCL2*, and *CXCR2* using publicly available 3′-end scRNA-seq dataset

**Fig. 7:**
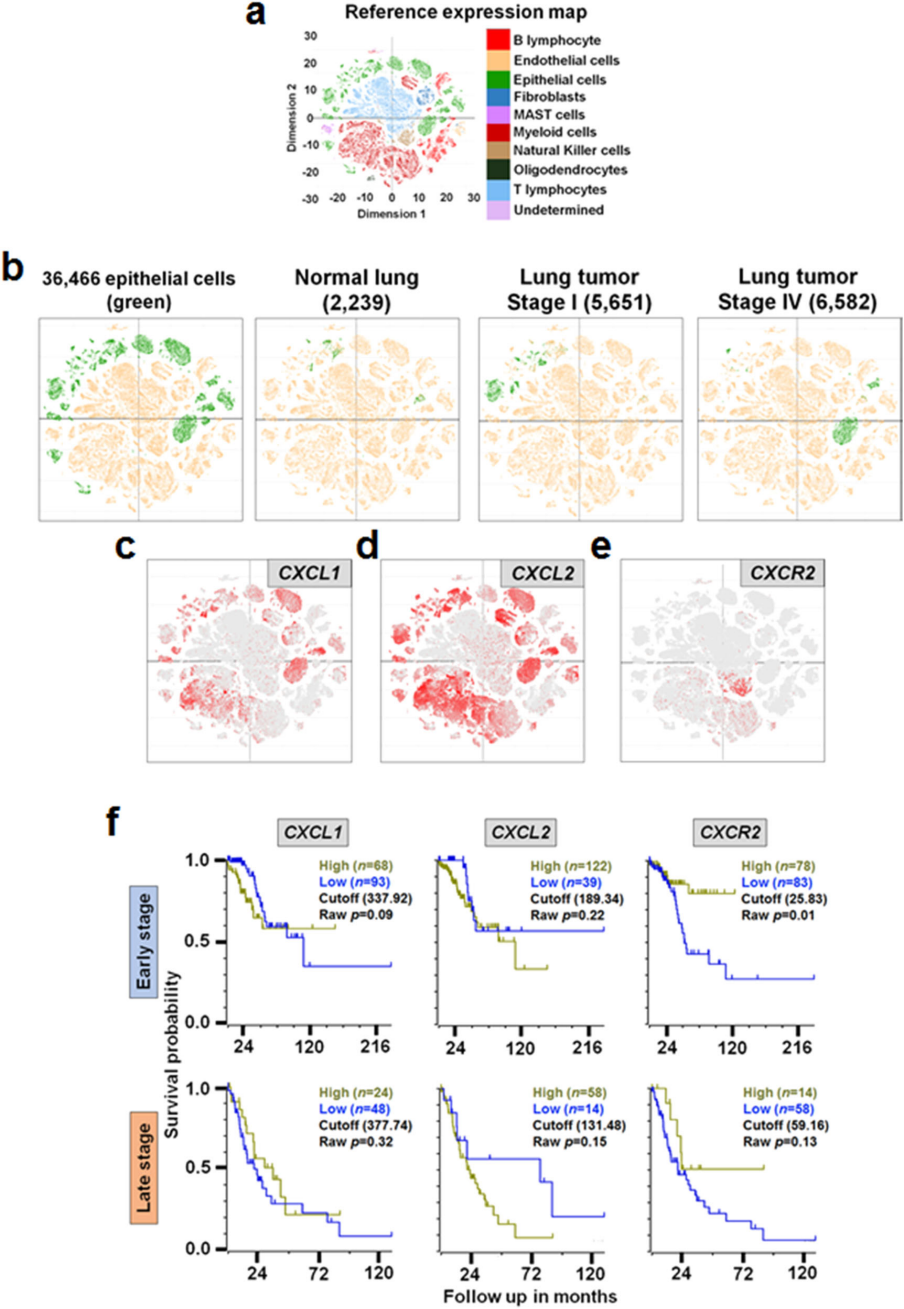
Validation of chemokine gene expression mined from public scRNA-seq dataset and survival analysis. **a** Reference expression map resulting from a clustering analysis of publicly available 10x Genomics 3′-end scRNA-seq dataset (GSE131907)^8^. The scRNA-seq dataset was generated from single cells (*n* = 208,506) isolated from multiple-stage LUAD patients (*n* = 58). **b** Four expression maps of epithelial cells (green) clustered from single cells of various tissues (normal and primary lung tumor tissues, normal and metastatic lymph nodes, metastatic brain tissues, and pleural fluids) of 58 LUAD patients at multiple disease stages (*n* = 36,466), only normal lung tissues at Stage I (*n* = 2,239), only primary lung tumors at Stage I (*n* = 5,651) and only primary lung tumors at Stage IV (*n* = 6,582) in the scRNA-seq dataset. **c**–**e** Expression maps of *CXCL1* in (**c**), *CXCL2* in (**d**), and *CXCR2* in (**e**) in various cell populations, including epithelial cells, from those LUAD patients at multiple disease stages. Refer to Supplementary Fig. 4 to identify clusters in a magnified reference expression map. **f** Kaplain–Meier lung cancer survival analysis plots based on high or low expression of *CXCL1*, *CXCL2*, and *CXCR2* in primary lung tumors profiled from bulk RNA-seq datasets of Stage I (*n* = 161) and Stage III & IV (*n* = 72) LUAD patients. *x* and *y* axes indicate follow-up in months and survival probability, respectively. The cut-off expression value between high and low expression per stage and gene was determined by a log-rank test.

To confirm the differential expression of the three chemokine genes profiled in the four human NSCLC epithelial cell lines that resulted from our 3′-end scRNA-seq analysis using Fluidigm C1 systems, we mined a publicly available 3′-end scRNA-seq dataset (GSE131907)^16^ that was sequenced from 208,506 single cells of normal and primary lung tumor tissues, normal and metastatic lymph nodes, metastatic brain tissues and pleural fluids of 58 LUAD patients at multiple disease stages in various cell populations. The dataset mined was generated from the Chromium Controller (10x Genomics, USA) (Hereafter 10x Genomics 3′-end scRNA-seq dataset to distinguish our Fluidigm 3′-end scRNA-seq dataset). From a clustering analysis of the 10x Genomics 3′-end scRNA-seq dataset (Fig. 7a), we identified that epithelial cells from normal lung and primary tumor tissues (Stage I and IV) were grouped into different clusters (Fig. 7b). These findings suggest a difference in gene expression at single-cell resolution in the epithelial cell populations between normal and primary tumor tissues, as well as early and late stages lung cancers. Specifically, the expression patterns of *CXCL1* (Fig. 7c), *CXCL2* (Fig. 7d), and *CXCR2* (Fig. 7e) in various cell populations, including epithelial cells, presented a positive correlation with our quantification result, that is, higher quantities of *CXCL2* than *CXCL1*, and relatively lower quantities of *CXCR2* than those of *CXCL1* and *CXCL2* in the 34 early-stage LUAD patients (Fig. 5 and Supplementary Fig. 2). We also performed a Kaplan–Meier survival analysis using publicly available 233 bulk RNA-seq datasets generated from primary tumors of 161 early-stage LUAD patients (Stage I) and 72 late-stage LUAD patients (Stage III and IV) (Fig. 7f). The survival plots revealed an inverse survival probability between the expression of *CXCL1* and *CXCL2* in primary tumors of early-stage LUAD patients. The high expression of *CXCR2* showed relatively higher survival probability at early stage compared to late-stage in primary tumors (Fig. 7f), regardless of a sex difference between female and male LUAD patients (Supplementary Fig. 5c). In addition, the high expression of *CXCL1* showed an inverse survival probability between female and male LUAD patients at early stage (Supplementary Fig. 5a), while the low expression of *CXCL2* was related to higher survival probability at late-stage of male LUAD patients up to 72 months (Supplementary Fig. 5b). The high and low expressions of chemokine and chemokine-receptor genes were determined at the cut-off expression values from a log-rank test.

### Protein expression validation

**Fig. 8:**
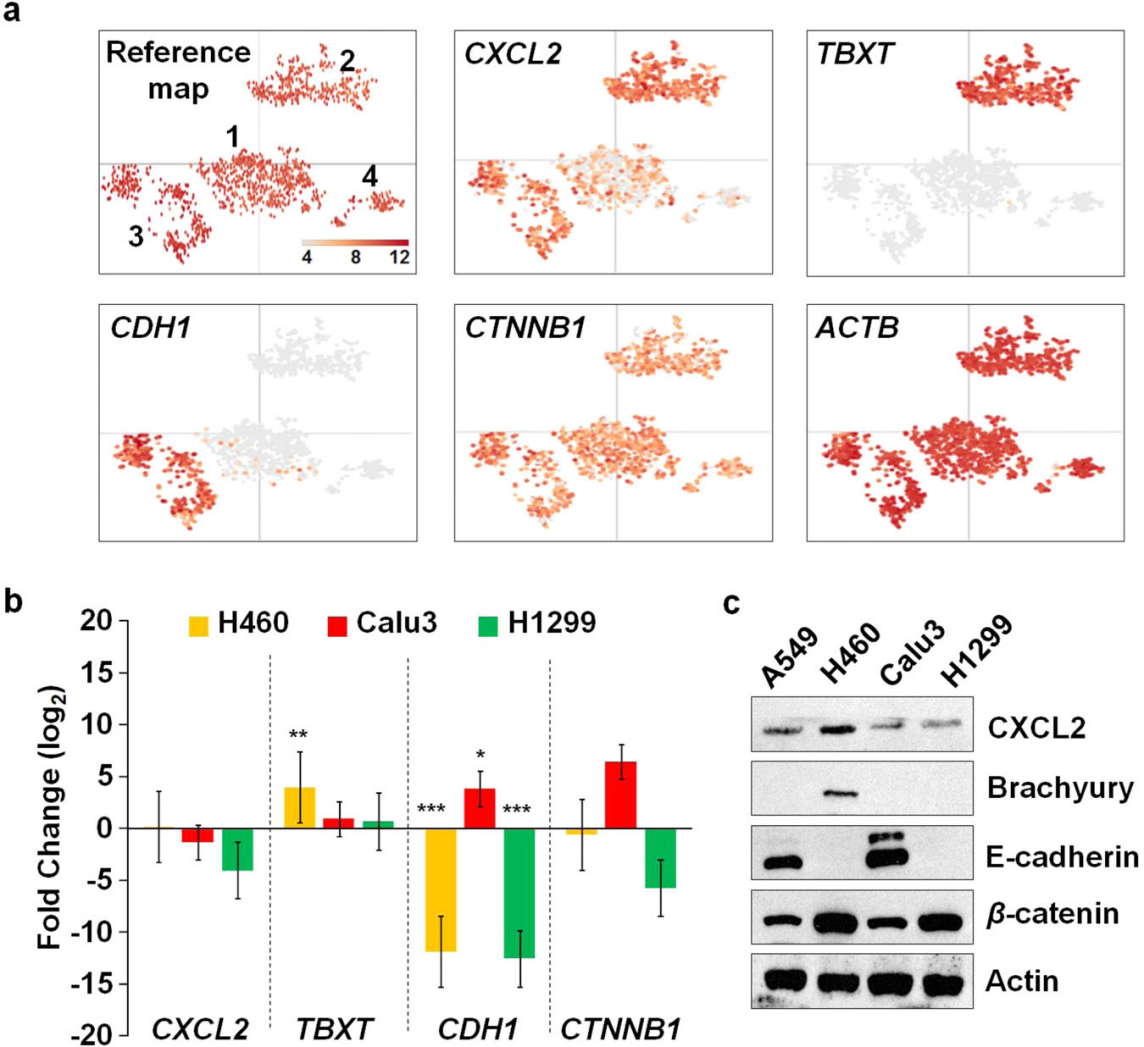
Validation of selected gene expression at protein level. **a** Expression maps presenting differential expression of 4 selected scRNA-seq DEGs (*CXCL2*, *TBXT*, *CDH1*, and *CTNNB1*) and *ACTB* (control gene) at single-cell resolution with a reference expression map. **b** qRT-PCR validation of fold-change values for the 4 selected DEGs at bulk-cell level; H460 (orange bars), Calu3 (red bars), and H1299 (green bars) compared with A549 (control cell line). *X* and *y* axes indicate gene names and fold-change values (log_2_), respectively. Fold-change values expressed as mean ± SEM from three separate experiments conducted in duplicate. Statistical significance; *P < 0.05, **P < 0.01, and ***P < 0.001. **c** Western blot analysis showing expression of proteins encoded by the 4 selected DEGs and Actin (loading control) in the four human NSCLC epithelial cell lines.

To extend our validation from gene to protein expression, we conducted western blot analysis to profile the expression of proteins encoded by 4 selected scRNA-seq DEGs. We chose *CXCL2*, T-box transcription factor T (*TBXT*), cadherin 1 (*CDH1*), and catenin beta 1 (*CTNNB1*), based on three criteria, (1) significant difference of fold-change values at single-cell level, (2) potentiality as a molecular biomarker in lung cancers, and (3) association with EMT. Differential expression of *CXCL2* was validated at single- and bulk-cell resolutions using Fluidigm 3′-end scRNA-seq and qRT-PCR analyses, respectively, in the four human NSCLC epithelial cell lines (Fig. 4a), 34 early-stage LUAD patients (Fig. 5b) and a various cell population of 58 multiple-stage LUAD patients using the 10x Genomics 3′-end scRNA-seq dataset (Fig. 7d). *TBXT* encodes brachyury that is associated with EMT in lung cancer^17^. The gene was ranked as the second-highest up-regulated gene in Cluster 2 against Cluster 1 (Fig. 4a). *CDH1* encoding E-cadherin was up-regulated (4.32x; Supplementary Data 1) in Cluster 3 against Cluster 1 and has been a known biomarker for EMT with *CTNNB1* (encoding beta catenin; β-catenin) in lung cancer^18^. First, the expression patterns of *CXCL2* in Fluidigm 3′-end scRNA-seq (Fig. 8a) and qRT-PCR datasets (Fig. 8b) were highly correlative with the protein expression of CXCL2 (Fig. 8c). *TBXT* was expressed in single cells >99% exclusively in Cluster 2 (Fig. 8a), thereby presenting significant up-regulation in H460 compared with A549 from a qRT-PCR analysis (Fig. 8b). Brachyury was also exclusively expressed in H460 (Fig. 8c) corresponding to the *TBXT* expression. The two EMT biomarkers, *CDH1* and *CTNNB1*, also showed positive correlation between gene (Fig. 8a and b) and protein levels (Fig. 8c). Overall, the expression pattern of the 4 selected scRNA-seq DEGs was positively correlated between 3′-end scRNA-seq (Fig. 8a) and qRT-PCR (Fig. 8b) analyses that was further validated by protein expression (Fig. 8c).

## Discussion

World-wide, lung cancer has the highest mortality rate amongst all cancers^1^. Although there are numerous explanations as to why this cancer is pervasive, a complex pattern of gene expression in individual tumor cells is a contributing factor, thereby making it difficult to characterize tumor transcriptomes. In our study, we employed a scRNA-seq application for the purpose of profiling DEGs at single-cell resolution in human NSCLC epithelial cells. We developed a robust scRNA-seq pipeline using the Fluidigm C1 systems that led to the identification of candidate genes causing transcriptomic complexity among individual cells in the lung cancer (Fig. 1d and Supplementary Data 1). In particular, a large portion of scRNA-seq DEGs were uniquely detected in the six gene sets (Fig. 2a), showing significantly high rates overlapping between scRNA-seq DEGs and intra-cell line DEGs (Fig. 2b). These confirm the power of our scRNA-seq application for discovering differential gene expression among individual lung cancer cells. Similarly, the significant overlapping rate between the DEGs of scRNA-seq and inter-cell lines (Fig. 2b) indicates that 3′-end scRNA-seq application can overcome limitation of traditional bulk RNA-seq for detecting intra-cell line DEGs.

From GSEA, we showed that diverse biological processes can be affected by differential expression of individual genes or gene families at single-cell resolution in NSCLC cells (Table 1 and Fig. 3). The GSEA with the up-regulated gene set in Cluster 4 against Cluster 1 revealed overrepresentation of relatively fewer GO terms or oncogenic gene sets with no enriched KEGG pathways when compared with enrichment results from GSEA with other five up- or down-regulated gene sets (Table 1). However, 2 of the 3 most enriched GO terms from the up-regulated gene set from Cluster 1 vs. Cluster 4 were associated with cell cycle (Fig. 3c), and there were another 8 cell cycle-associated GO terms enriched from the GSEA with the down-regulated gene set in Cluster 3 against Cluster 1 (Supplementary Data 2). For example, cyclin D gene family members presented differential expression at single-cell resolution among four clusters (Supplementary Fig. 6c), and we validated a down-regulation of cyclin D1 (*CCND1*) in H460 compared with A549 (Supplementary Fig. 6a). Previous work by others reported a decrease in the expressions of *CCND1* and protein product, cyclin D, in H460^17^. Cyclin D-CDK complex progresses cells from G0/G1 to S phases of cell cycle in downstream of RAS-Phosphoinositide 3 kinase-AKT serine/threonine kinase (RAS-PI3K-AKT) signaling pathway^19^. Others have found a significant overrepresentation of RAS and PI3K signaling pathways through a GSEA with up-regulated genes in brachyury-knockdown MDA-MB-231 cells compared with MDA-MB-231 control cells^20^. With our scRNA-seq DEG dataset from human NSCLC epithelial cells, we also identified corresponding expression patterns for genes involved in the RAS-PI3K-AKT signaling pathway (Supplementary Fig. 7). Here, there were 12 down-regulated and 10 up-regulated genes in Cluster 2 (comprised of cells from H460 that predominantly express brachyury) against Cluster 1 (comprised of cells from predominantly brachyury-null A549, Calu3, and H1299) (Supplementary Fig. 7a). We also found 10 down-regulated and 44 up-regulated genes in Cluster 3 (comprised of cells from predominantly brachyury-null Calu3) against Cluster 1 in the signaling pathway (Supplementary Fig. 7b). *KRAS* is a RAS family member that is frequently (~85%) mutated in cancers more than any other family member, namely NRAS (~15%) and HRAS (<1%)^21^. A549 and H460 NSCLC cell lines express non-synonymous substitution of *KRAS*, while the gene is wild type in Calu3 and H1299 NSCLC cell lines^9^. Thus, the identification of relatively more down-regulated genes in Cluster 2 compared with those in Cluster 3 against Cluster 1 in the RAS-PI3K-AKT signaling pathway indicates that a reduced expression level of cell cycle-associated genes may be correlated with the presence of brachyury in *KRAS*-mutated NSCLC cells. Alternatively, brachyury overexpression and single-point mutation on *TBXT* are transcriptomic and genomic characteristics for chordoma diagnosis^22^, where one of the primary genetic alternations is frequent mutation (homozygous deletion) on *CDKN2A* gene encoding p16 protein^23^. As a tumor suppressor, p16 regulates the cyclin D-CDK complex. However, p16 is oncogenic when unable to form a complex with cyclin D-CDK due to mutations that prevent p16 binding^24^. In lung cancer, homozygous deletion of *CDKN2A* is often found as in chordomas^25^. Although *CDKN2A* was preferentially expressed in Calu3 and H1299 cells (Fig. 3c), p16 is dysfunctional in all four NSCLC cell lines used in the current study because of mutations in *CDKN2A*^9^. Previous studies by others reported that co-mutations on *KRAS*, *CDKN2A,* and/or *CDKN2B* (encoding p15 protein) accelerated tumorigenesis in mouse lung^26^ and human lung cancer patients at early stage^27^. These findings suggest that brachyury may regulate cell-cycle progression in brachyury-expressing cells where *KRAS* and *CDKN2A* are co-mutated. Therefore, a differential expression of cell cycle-related genes may be one of the factors to contribute to an increase in transcriptomic complexity at single-cell resolution in human NSCLC epithelial cells.

In cancers, chemokines facilitate inflammatory events by recruiting immune cells to tumor sites, thus supporting metastasis and tumorigenesis^28^. Because of these functions, it is suggested that chemokines may service as a cancer diagnostic biomarker^29^ and viable candidate for targeted immunotherapy^30^. In lung cancer, previous studies by others reported that NSCLC patients showed higher concentration of CXCL2 when compared with that of the protein in chronic obstructive pulmonary disease patients^31^. More recently, it was reported that Cxcl2 secretion increased in intra-tumor cells of *Kras*-mutated lung cancer mouse model, likely contributing to immune escape process of lung cancer cells^32^. In our study, we observed a significant up-regulation of most *CXCL* gene family members in Cluster 2 and Cluster 3 compared with Cluster 1 at single-cell level (dark-purple bars; Fig. 4a and b, respectively) as well as H460 and Calu3 compared with A549 at bulk-cell level (dark-green bars; Fig. 4a and b, respectively) in NSCLC cells. This strongly suggested that chemokine-encoding genes may present a quantitative difference in lung cancers, and if so, there may be epigenetic regulators associated with a transcriptional process of chemokine mRNAs. Therefore, we quantified mRNAs of *CXCL1*, *CXCL2*, and *CXCR2* and associated epigenetic regulators, miR-532-5p, miR-1266-3p, and miR-3163, respectively, in primary lung tumors and normal lung tissues of 34 early-stage LUAD patients and finally found the inverse correlation between the quantities of chemokine mRNAs and the copy numbers of corresponding microRNAs (Fig. 5). Interestingly, the inverse correlation was more clearly observed in early-stage male LUAD patients (Fig. 5). The quantitative differences in chemokine gene expression that were validated in four human NSCLC epithelial cell lines (Fig. 4) and 34 early-stage LUAD patients (Fig. 5 and Supplementary Fig. 2) were additionally supported by the publicly available 10x Genomics LUAD 3′-end scRNA-seq dataset (Fig. 7c–e). These findings suggest further transcriptomic characterization of chemokine genes and epigenetic transcription regulators is warranted at single-cell resolution in primary tumors between the early- and late-stage LUADs.

MicroRNAs are abundant in human liquid biopsies^33^, thus quantification of miRNA copy numbers from blood (plasma) can be an effective strategy for identifying aberrant gene expression from various stages of cancer and developing diagnostic molecular biomarkers. In our study, we showed the inverse correlation between chemokine mRNAs and epigenetic regulatory microRNAs in primary lung tumors against normal lung tissues of 34 early-stage LUAD patients (Fig. 5 and Supplementary Fig. 3). At the time of surgical resection, blood was harvested from those patients after which plasma was isolated to determine whether miR-532-5p, miR-1266-3p, and miR-3163 can be applicable as a molecular biomarker for diagnose of lung cancers using liquid biopsy samples. Here, we discovered a higher copy number of miR-532-5p (Fig. 6a), post-transcriptionally regulating *CXCL1* and/or *CXCL2*, compared with those of miR-1266-3p (Fig. 6b) and miR-3163 (Fig. 6c) in plasma of the 34 early-stage LUAD patients. In agreement with our work, recent studies by others reported that miR-532-5p functions as a tumor suppressor in tongue^34^, renal^35^, ovarian^36^, and lung cancers^37^. Further characterization of miR-532-5p will contribute to a better understanding of its biological functions, including post-transcriptional regulatory processes of chemokine genes, thereby helping establish viable biomarkers that are accessible by non-invasive liquid biopsies for detection of lung cancer.

In the current study, we successfully profiled the expression of *CXCL2* at single- and bulk-cell levels in four human NSCLC epithelial cell lines using Fluidigm 3′-end scRNA-seq and qRT-PCR analyses (Fig. 4a). We also showed the quantitative differences of the gene between primary lung tumors and normal lung tissues from early-stage LUAD patients (Fig. 5b) and confirmed the differential expression of *CXCL2* in diverse cell populations, including epithelial cells, from multiple-stage LUAD patients (Fig. 7d). Finally, we showed positive correlation between the expression of *CXCL2* mRNAs and CXCL2 proteins in human NSCLC epithelial cells (Fig. 8c). Of the selected lung cancer-related EMT genes, *TBXT* was exclusively expressed in Cluster 2 (Fig. 8a) and H460 (Fig. 8b) at both gene and protein (brachyury) levels (Fig. 8c). Previous work by others reported that, in the presence of brachyury, expression of E-cadherin (*CDH1*) was down-regulated, thereby promoting EMT in lung cancer cells^17^. As part of the E-cadherin complex, β-catenin (*CTNNB1*) plays a role in anchoring the inner membrane of a cell for cell–cell adhesion^38^, and its expression has been used as an EMT biomarker^39^. In our study, we discovered a positive correlation between the expression of *CDH1* and *CTTNB1* at bulk-cell level (Fig. 8b) in human NSCLC epithelial cells. The expressions of *CDH1* (Fig. 8b) and E-cadherin (Fig. 8c) were also positively correlated at bulk-cell level, while the expression pattern of *CTNNB1* at single-cell level (Fig. 8a) showed more similar with that of β-catenin (Fig. 8c) compared with their expressions at bulk-cell level (Fig. 8b) in the four NSCLC epithelial cell lines. Furthermore, others reported that brachyury expression in H460 is positively correlated with CXCL8 and CXCR2, and CXCL8-CXCR2 complex presumably leads to enhanced EMT^17^. We found that *CXCL8* was up-regulated in Cluster 2 (Fig. 4a) comprised of cells predominantly from *TBXT* (Brachyury)-expressing H460 (Fig. 8) when compared to Cluster 1 where most cells were from *TBXT* (Brachyury)-null cells lines (Fig. 8). However, there was no evidence of a significant difference in *CXCR2* expression at the single-cell level among four clusters as well as at bulk-cell level amongst the four human NSCLC epithelial cell lines (data is not shown) and 34 early-stage LUAD patients (Fig. 5c). We did however identify alteration in *CXCR2* expression at single-cell resolution in natural killer and myeloid cell populations from multiple-stage LUAD patients (Fig. 7e), and a relatively less significant correlation between survival probability in primary tumors of late-stage LUAD and the high expression of *CXCR2* when compared to the high *CXCR2* expression in primary tumors of early-stage LUAD (Fig. 7f). Compared with work by others that described elevated expression of CXCR2 in infiltrating neutrophils from microarrayed Ras-driven LUAD to be associated with poor prognosis^40^, the relatively lower quantity of circulating miR-3163 (Fig. 6c) that regulates an expression of *CXCR2* mRNAs compared with that of plasma-isolated miR-532-5p and miR-1266-3p regulating *CXCL1* and *CXCL2* mRNAs, respectively, suggests further work is required to understand how chemokine/chemokine-receptor expression in various cell populations within a given LUAD relates to disease progression, recurrence and response to treatment.

In conclusion, we successfully established scRNA-seq pipeline using the Fluidigm C1 systems and demonstrated that our scRNA-seq workflow is highly robust for detecting and profiling differential gene expression at single-cell resolution in lung cancers. More specifically, experimental and bioinformatic validation of chemokine gene quantity and copy number of corresponding microRNAs in solid and liquid LUAD patient samples confirms that a quantitative difference in the chemokine gene mRNAs and corresponding microRNAs can be used as molecular signatures for characterizing lung cancers. In our current study, unique transcriptomic characteristics that we elucidated at the single-cell resolution will provide a framework for the development of early-stage diagnostic biomarkers, thus advancing strategies for improving precision medicines for the treatment of lung cancers.

## Methods

### Cell lines

Four human NSCLC epithelial cell lines, A549 (ATCC-CCL-185), H460 (ATCC-HTB-177), Calu3 (ATCC-HTB-55), and H1299 (ATCC-CRL-5803), were purchased from ATCC (VA, USA) and used immediately for a 3′-end scRNA-seq analysis. Cells were sieved through 40 µm cell strainers followed by live-cell collection using EasySep^TM^ Dead Cell Removal (Annexin V) kits (STEMCELL Technologies, Inc.). The number and viability of selected cells were assessed using TC20™ Automated Cell Counter systems (Bio-Rad Laboratories, Inc.), and then cells were adjusted at the concentration of 400 cells/µL per cell line in media comprised of phosphate-buffered saline (PBS; Gibco), 5% (v/v) of fetal bovine serum (FBS; Gibco), and 1 mM of calcium chloride.

### Construction of dual-indexed and 3′-end enriched cDNA libraries for NGS

For successful downstream analyses, it is essential to capture one single cell per chamber in integrated fluidic circuits (IFCs; Fluidigm, Inc.). To achieve this, cell-buoyancy tests were performed at five different titrations with the volume-to-volume (v/v) ratio of 1:9, 9:1, 7:3, 6:4, and 5:5 between cells and C1 suspension reagent (Fluidigm, Inc.) followed by visual examination using an inverted microscope. We selected the buoyancy ratio (v/v) of 5.5 (cells) to 4.5 (C1 suspension reagent). Four thousand epithelial cells (10–17 μm) per cell line were loaded to an IFC (PN 101-4964; Fluidigm, Inc.), after which single cells captured in individual chambers of IFCs were manually confirmed using an inverted microscope and scored on the C1 high-throughput (HT) workbook (PN 101­5976; Fluidigm, Inc.). NGS datasets obtained from only single cell-containing IFC chambers were used for the downstream bioinformatic analyses. Cell lysis, total RNA isolation, cDNA synthesis, and pre-amplification of synthesized cDNAs were performed using the C1 script of ‘mRNA Seq HT:RT & Amp (1912x)’ (Fluidigm, Inc.). During cDNA pre-amplification, individual cells were pre-barcoded at 3′-end cDNAs with 40 different Fluidigm cell-specific indexes. Following the completion of pre-amplified cDNA preparation in the C1 systems, individual pre-indexed cDNA samples, including External RNA Controls Consortium (ERCC) spike-in (Invitrogen) that was pre-diluted at the ratio of 1:60,000, were transferred from an IFC to a regular 96-well PCR plate. For dual-indexing, 20 different i7 barcode-containing primers in Nextera XT index Kit v2 Set A/B (Illumina, Inc.) were annealed to 5′-end of fragmented cDNAs following tagmentation of individual cDNA samples. We constructed a total of 1,600 dual-indexed and 3′-end enriched cDNA libraries (400 cDNA libraries per cell line) using Nextera XT DNA library preparation kits (Illumina, Inc.). Ten cDNA library pools (40 libraries per cDNA library pool) per cell line were individually quantified using Qubit assay (Invitrogen), and further quality and quantity check of the cDNA library pools was performed in 2100 Bioanalyzer systems (Agilent, Inc.). Based on the molarity measured, individual cDNA library pools were equimolarly combined and sequenced in the Center for Gastrointestinal Biology and Disease (NC, USA) using NextSeq 500 systems (Illumina, Inc.). We used the C1 mRNA Sequencing High Throughput Demultiplexer Script (https://www.fluidigm.com/software) and Geneious Prime 2019.2.1 (https://www.geneious.com) to demultiplex individual NGS read sets and deposited the demultiplexed datasets to NCBI GEO database (GSE183590).

### Bioinformatic analysis

BBDuk Trimmer (a part of Bestus Bioinformatics Tools; RRID:SCR_016968) was used to process individual demultiplexed raw NGS read sets by trimming out low-quality reads at quality score <10^−3^ (equivalent to Phred score 30 indicating the sequencing-error rate at one base per 1000 bases) and adapter/primer sequence-contaminated reads at read length >30 bases. We employed HISAT2 (RRID:SCR_015530) for read mapping to align processed reads to human reference genome (GRCh38.p13). Aligned reads per gene were quantified using FeatureCounts (A part of Subread; RRID:SCR_009803). Next, we prepared a data matrix comprised of read counts per gene (row) in each cell (column), and the data matrix prepared was imported to a single-cell analysis tool, ASAP^41^, to normalize gene expression values, reduce dimensionality of highly variable normalized expression values per gene, re-arrange individual cells by a clustering analysis and finally detect DEGs by a comparison of generated clusters. To obtain normalized expression values per gene, we applied ‘Counts per Million (CPM)’ as a scaling factor at CPM per gene ≥1 using a read-scaling tool, voom^42^. A t-SNE analysis was carried out for non-linear dimensionality reduction of highly complex and variable normalized gene expression datasets. Following a clustering analysis with the SC3 clustering tool, we assigned Cluster 1 (Fig. 1b) as a control cluster and individually compared normalized expression values per gene in single cells of Cluster 1 with those in single cells of other three clusters to identify DEGs using a DEG detection tool, limma (RRID:SCR_010943). For downstream validation, we prioritized DEGs that were mapped with reads per gene ≥4 and fulfilled with fold-change differences ≥|2| at the statistical significance of FDR-corrected *P* value < 0.05.

### Gene expression profiling

For a hierarchical heatmap-clustering analysis, the read-count data matrix (read counts/gene/individual cells) were imported to Morpheus by Broad Institute (RRID:SCR_017386). The data matrix was then adjusted in the range of Z-score from −1.50 (lowest expression) to 1.50 (highest expression). Furthermore, we prepared six different DEG sets comprised of up- or down-regulated genes from Cluster 1 vs. Cluster 2, 3, and 4 (two gene sets per cluster comparison) that included DEGs commonly detected from more than two cluster comparisons. The six DEG sets prepared were used as inputs to create volcano plots per cluster comparison with the GraphPad Prism (RRID:SCR_002798) and were used to identify (1) uniquely up- or down-regulated genes per cluster comparison and (2) GO terms in the category of biological processes overrepresented with an up- or down-regulated gene set per cluster comparison using a GSEA tool, PANTHER (RRID:SCR_004869). In the ASAP, we used those six DEG sets for GSEA on KEGG pathways and the 189 oncogenic gene sets available in KEGG (RRID:SCR_012773) and Molecular Signatures (RRID:SCR_016863) databases, respectively. We set the cut-off of FDR-corrected *P* value < 0.05 to obtain statistically confident enrichment values from each GSEA.

### Patient primary lung tumors

The authors declare patient materials were obtained in compliance with the Nova Scotia Health Authority, and all experiments were approved with written consent under the Nova Scotia Health Authority REB # 1024460 guidelines. All LUADs and normal lung tissues described in our study were prepared for validation immediately upon receipt from the surgical suite.

### Experimental validation of fold-change values using a qRT-PCR analysis

TRIzol^TM^ Reagent (Invitrogen) was used to isolate total RNAs from the four human NSCLC epithelial cell lines and paired primary lung tumor and normal lung tissues from 34 Stage I LUAD patients (16 female and 18 male). TURBO^TM^ DNA-free kits (Invitrogen) were used to remove residual genomic DNAs in isolated total RNAs. Total RNAs were quantified using DU 800 UV spectrophotometer systems (Beckman Coulter, Inc.). Once quantified, 2 and 1 µg of total RNAs per cell line and LUAD patient sample, respectively, were used to synthesize single-strand cDNAs with QuantiTect Reverse Transcription kits (Qiagen, Inc.) for two-step qRT-PCR analysis. The synthesized single-strand cDNAs of 100 and 50 ng per cell line and LUAD patient sample, respectively, were utilized as a template for qPCR analyses with QuantiFast SYBR green PCR kits (Qiagen, Inc.) in AriaMx qPCR systems (Agilent, Inc.) following manufacturer’s instruction of the qPCR kits. Primer pairs for qPCR analyses were selected from previous studies by others or either of ‘PrimerBank’ (RRID:SCR_006898) or ‘RTPrimerDB-The Real-Time PCR and Probe Database’ (RRID:SCR_007106). When needed, custom primer pairs were designed using Primer3 (RRID:SCR_003139). For information on primer pairs used in the current study see Supplementary Data 6. All qPCRs were performed on two replicates per cDNA sample and repeated three times per gene of interest. To obtain the fold-change values of 120 selected DEGs in NSCLC cell lines using a qRT-PCR analysis, we applied the Pfaffl’s method^43^ to normalize raw Ct values using A549 and actin gamma 1 (*ACTG1*) as a control sample and endogenous reference gene, respectively.

### Absolute quantification of selected chemokine mRNAs and microRNA copy numbers in LUAD patient samples

For qRT-PCR quantification, we applied a standard curve approach to measure quantities of three chemokine mRNAs, *CXCL1*, *CXCL2*, and *CXCR2*, and the corresponding three microRNAs, miR-532-5p, miR-1266-3p, and miR-3163, in cDNAs synthesized from total RNAs isolated from primary lung tumors and normal lung tissues resected from surgery of 34 Stage I LUAD patients with no prior treatment. The three microRNAs were selected from miRDB (RRID:SCR_010848) based on sequence similarity in 3′-untranslated regions (UTRs) (Supplementary Data 6). For a qRT-PCR analysis to measure microRNA copy numbers in liquid (plasma) biopsy samples, cell-free total RNAs were isolated from 200 μL per plasma sample of the corresponding early-stage LUAD patients (Supplementary Data 5) using miRNeasy Serum/Plasma Advanced kits (Qiagen, Inc.). Following genomic DNA removal with TURBO^TM^ DNA-free kits, single-strand cDNAs per plasma sample were synthesized using Mir-X^TM^ miRNA First-Strand Synthesis kits (Takara Bio USA, Inc.) following manufacturer’s instruction of the kits. For absolute quantification, standard curves were prepared (copy number range from 1 × 10^6^ to 8 × 10^3^) with 5x serial dilutions of single-strand cDNAs synthesized from cel-miR-39 miRNA mimic included in miRNeasy Serum/Plasma Spike-In Control kits (Qiagen, Inc.) following manufacturer’s instruction of the kits. qPCRs were performed on two replicates per sample and repeated three times/microRNA using TB Green Advantage qPCR Premix kits (Takara Bio USA, Inc.) in AriaMx qPCR systems (Agilent, Inc.). U6 snRNAs were also used as an endogenous reference gene to obtain an averaged-correction factor per qPCR array and normalize raw Ct values, thereby calculating copy numbers by 10^((normalized Ct value of targeting miRNA – intercept at *y* axis)/-slope)^. The values of intercept at *y* axis and slope per qPCR array were obtained from the standard curves prepared with the cel-miR-39 spike-in control.

### Bioinformatic mining of publicly available LUAD 10x Genomics 3′-end scRNA-seq dataset

We mined publicly available LUAD 10x Genomics 3′-end scRNA-seq dataset (GSE131907) to validate differential expression of genes profiled with our Fluidigm 3′-end scRNA-seq and qRT-PCR datasets from the four NSCLC cell lines and 34 Stage I LUAD patient samples, respectively. Whole read-count data matrix was imported to the ASAP single-cell analysis tool. The read-count data matrix of whole-cell populations was used to identify clusters that contain epithelial cells from (1) normal lung and primary tumor tissues at multiple stages, (2) normal lung at Stage I, (3) primary tumors at Stage I, and (4) primary tumors at Stage IV. In addition, a Kaplan–Meier survival analysis was performed to investigate correlation between the high or low expression level of the three chemokine genes and survival probability of female and male LUAD patients at Stage I (early) and Stage III & IV (late). To create survival plots per gene, stage, and sex, we used the R2 genomics analysis and visualization platform (https://hgserver1.amc.nl/cgi-bin/r2/main.cgi) that contains bulk RNA-seq datasets generated by The Cancer Genome Atlas (TCGA) program (RRID:SCR_003193) publicly available in the Genomic Data Commons Data Portal (RRID:SCR_014514).

### Western blotting analysis

Proteins were isolated from the four human NSCLC epithelial cell lines using cell lysis buffer as previously described^44^. Quantification of proteins in cell lysates was conducted by Bradford Assay followed by SDS-PAGE gel electrophoresis. The following primary antibodies diluted were used for western blot analyses^44^: (1) CXCL2 (1:1,000; Cat. No. ab91511, Abcam); (2) brachyury (1:30,000; Cat. No. 81694, Cell Signaling Technology); (3) E-cadherin (1:1,000; Cat. No. 3195, Cell Signaling Technology); (4) β-catenin (1:2,000; Cat. No. 9562, Cell Signaling Technology); and (5) actin (1:2,000; Cat. No. ab8229, Abcam). Proteins were visualized by chemiluminescence autoradiography.

### Statistics

Statistical significance for absolute and relative quantifications in qRT-PCR analyses was determined using two-sample *t*-tests and non-parametric Wilcoxon Signed Rank Test at two-sided *P* value < 0.05, respectively. Linear regression analyses were performed to identify coefficient of determination (*r*^2^) for fold-change values of DEGs detected from Fluidigm 3′-end scRNA-seq and qRT-PCR analyses at the statistical significance of *P* value < 0.0001. One-way ANOVA was applied to determine statistical significance at *P* value < 0.05 for three repeated measurements of microRNA copy numbers in plasma samples. All the data are expressed as the standard error of the mean (SEM) from three repeated experiments using duplicated samples.

### Reporting summary

Further information on research design is available in the Nature Research Reporting Summary linked to this article.

## Supplementary information

Supplementary Information

Supplementary Data 1

Supplementary Data 2

Supplementary Data 3

Supplementary Data 4

Supplementary Data 5

Supplementary Data 6

Reporting Summary

## Data Availability

scRNA-seq demultiplexed datasets that support the findings of this study have been deposited to NCBI GEO database with the accession number GSE183590.
