# Supplementary material for: Single-cell RNA sequencing for the identification of early-stage lung cancer biomarkers from circulating blood
Source: NPJ Genom Med. 2021 Oct 15;6:87. doi: 10.1038/s41525-021-00248-y (PMC8519939; doi:10.1038/s41525-021-00248-y)
Supplement: Supplementary file 1 — Supplementary Information [file 41525_2021_248_MOESM1_ESM.pdf]

Supplementary Table 1. Information on 3'-end scRNA-seq dataset used for gene expression profiling analysis.

Cell line	Raw data from NGS		After processing raw data		After read mapping with processed data	
	No. of cells	No. of reads	No. of cells (%)[a]	No. of reads (%)[b]	No. of cells[c] (%)	No. of reads (%)
**A549**	400	44,009,436	391 (97.8)	25,708,488 (58.4)	391 (97.8)	20,016,549 (45.5)
**H460**	400	45,299,075	383 (95.8)	26,463,657 (58.4)	381 (95.3)	20,455,852 (45.2)
**H1299**	400	42,205,130	328 (82.0)	24,391,885 (57.8)	319 (79.8)	19,780,315 (46.9)
**Calu3**	400	48,437,279	354 (88.5)	29,925,212 (61.8)	350 (87.5)	22,678,217 (46.8)
**Total (%)**	1,600	179,950,920	1,456 (91.0)	106,489,242 (59.2)	1,441 (90.1)	82,930,933 (46.1)

[a, b]Against raw data and [c]15 read sets (or cells) containing <2,000 sequence reads were excluded after processing raw read sets.

Supplementary Table 2. The number of single cells regrouped from four human NSCLC epithelial cell lines into four clusters.

Clusters / Cell lines (%)[a]	A549	H460	Calu3	H1299	Total per cluster (%)[c]
**Cluster 1**	317 (52.0)	77 (12.6)	46 (7.5)	170 (27.9)	610 (42.3)
**Cluster 2**	74 (19.6)	304 (80.4)	0	0	378 (26.2)
**Cluster 3**	0	0	277 (80.3)	68 (19.7)	345 (23.9)
**Cluster 4**	0	0	27 (25.0)	81 (75.0)	108 (7.5)
**Total per cell line (%)[b]**	391 (27.1)	381 (26.4)	350 (24.3)	319 (22.1)	1,441

[a]Against the number of single cells per cluster and [b,c]against the total number of 1,441 single cells.

Supplementary Table 3. The number of GO terms, KEGG pathways and Molecular Signatures (oncogenic gene sets) overrepresented from GSEA.

Gene set (Cluster)[a]	No. of DEGs	No. of GO terms[b]	No. of pathways	No. of signatures
**Up (Cluster 2)**	355	55	6	18
**Down (Cluster 2)**	305	40	0	12
**Up (Cluster 3)**	939	161	26	38
**Down (Cluster 3)**	1,172	157	51	27
**Up (Cluster 4)**	199	3	0	3
**Down (Cluster 4)**	226	68	6	15
**Total[c]**	2,655	441	79	72

[a]Against Cluster 1, [b]including overlapping GO terms and [c]unique DEGs or GO terms.

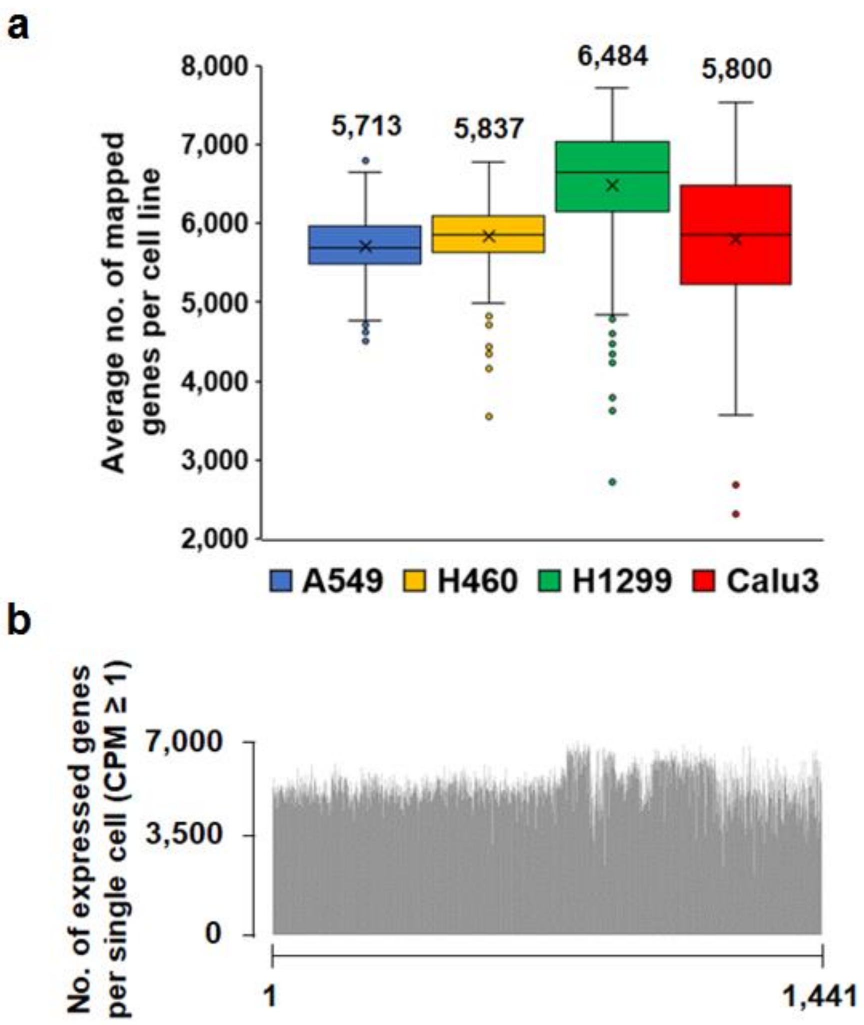
a
Average no. of mapped genes per cell line
8,000
7,000
6,000
5,000
4,000
3,000
2,000
5,713
5,837
6,484
5,800
A549
H460
H1299
Calu3
b
No. of expressed genes per single cell (CPM ≥ 1)
7,000
3,500
0
1
1,441

Kim *et al.*, Supplementary Figure 2

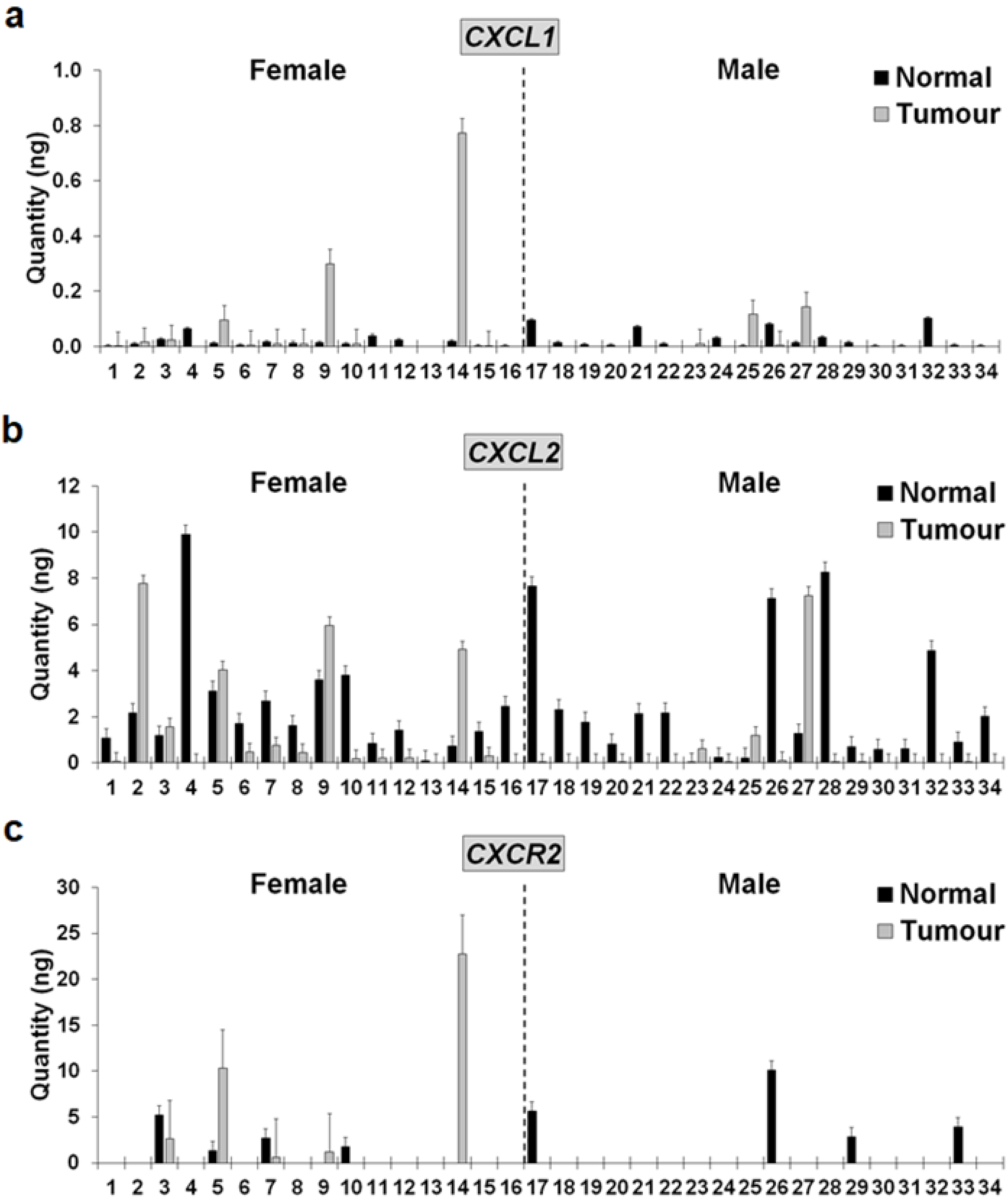

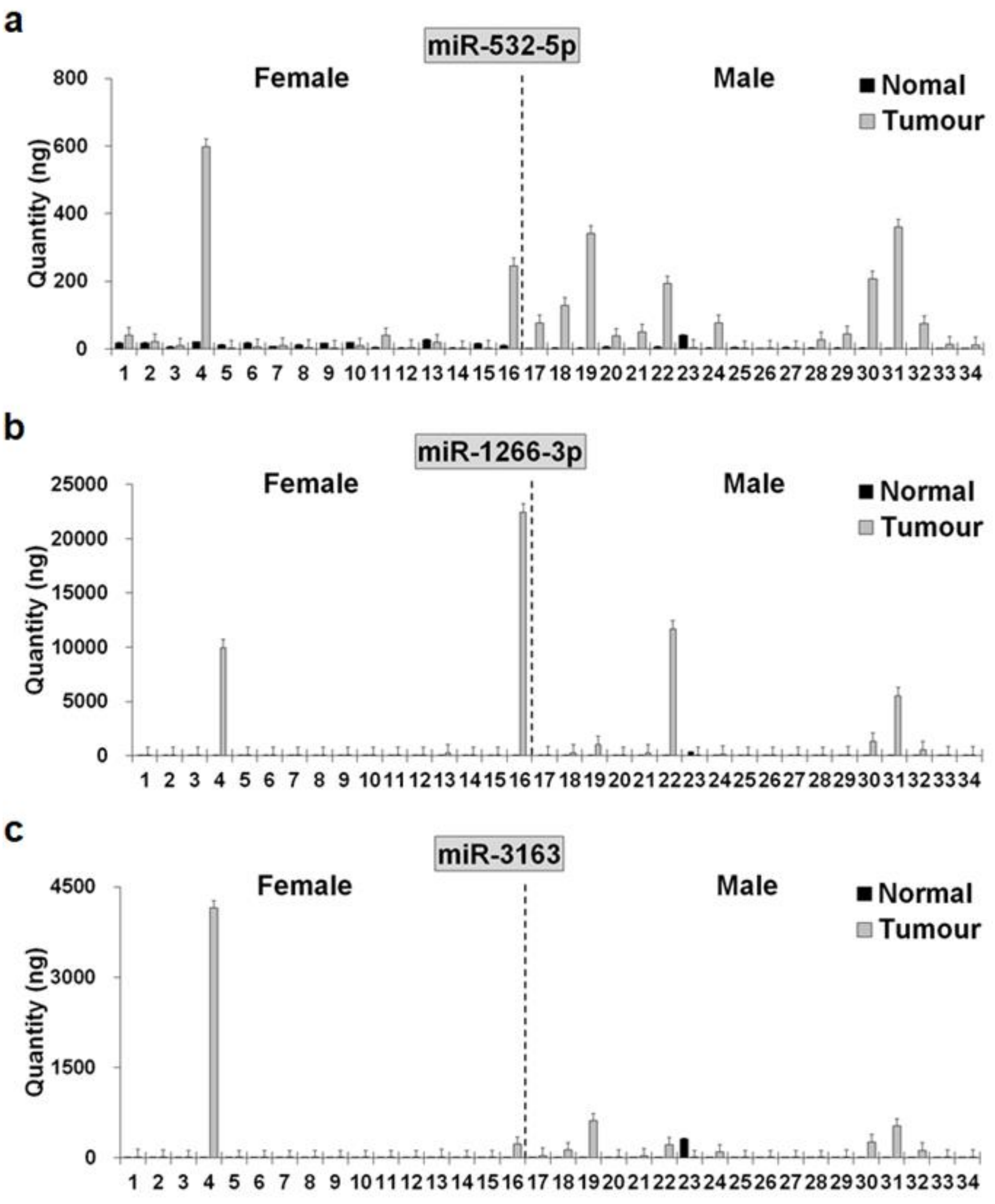
a
miR-532-5p
Female
Male
Nomal
Tumour
Quantity (ng)
0
200
400
600
800
1 2 3 4 5 6 7 8 9 10 11 12 13 14 15 16 17 18 19 20 21 22 23 24 25 26 27 28 29 30 31 32 33 34
b
miR-1266-3p
Female
Male
Normal
Tumour
Quantity (ng)
0
5000
10000
15000
20000
25000
1 2 3 4 5 6 7 8 9 10 11 12 13 14 15 16 17 18 19 20 21 22 23 24 25 26 27 28 29 30 31 32 33 34
c
miR-3163
Female
Male
Normal
Tumour
Quantity (ng)
0
1500
3000
4500
1 2 3 4 5 6 7 8 9 10 11 12 13 14 15 16 17 18 19 20 21 22 23 24 25 26 27 28 29 30 31 32 33 34

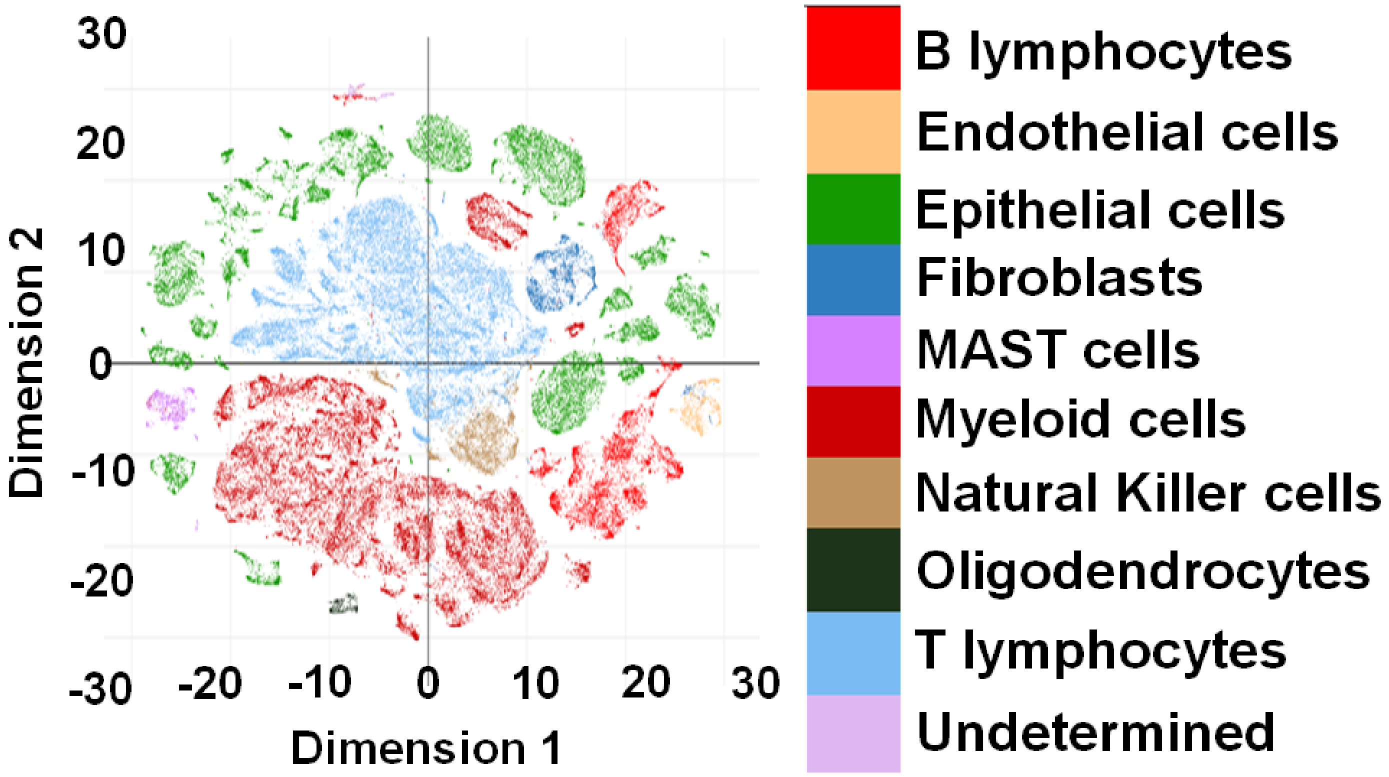

30
20
10
0
-10
-20
-30
-20
-10
0
10
20
30
Dimension 2
Dimension 1
B lymphocytes
Endothelial cells
Epithelial cells
Fibroblasts
MAST cells
Myeloid cells
Natural Killer cells
Oligodendrocytes
T lymphocytes
Undetermined

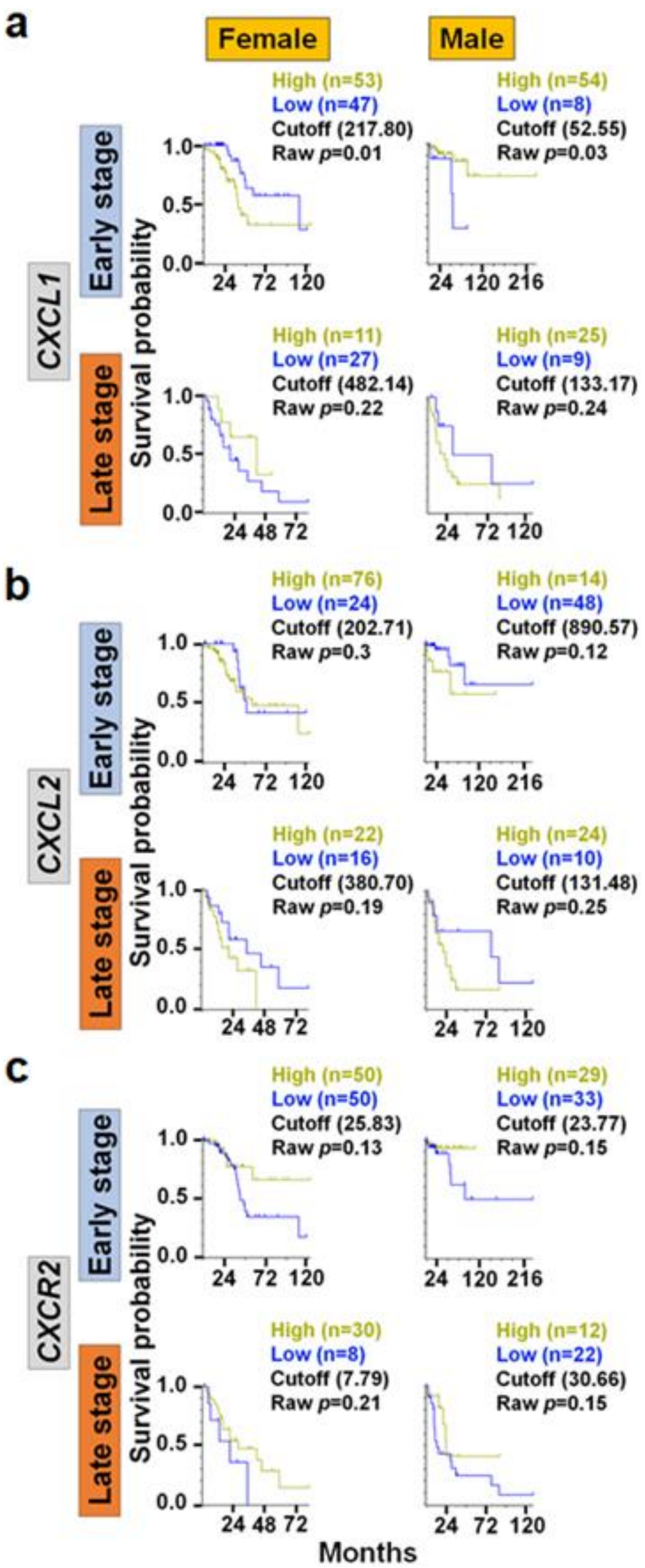
a
Female
Male
CXCL1
Early stage
Late stage
Survival probability
High (n=53)
Low (n=47)
Cutoff (217.80)
Raw p=0.01
High (n=54)
Low (n=8)
Cutoff (52.55)
Raw p=0.03
High (n=11)
Low (n=27)
Cutoff (482.14)
Raw p=0.22
High (n=25)
Low (n=9)
Cutoff (133.17)
Raw p=0.24
b
CXCL2
High (n=76)
Low (n=24)
Cutoff (202.71)
Raw p=0.3
High (n=14)
Low (n=48)
Cutoff (890.57)
Raw p=0.12
High (n=22)
Low (n=16)
Cutoff (380.70)
Raw p=0.19
High (n=24)
Low (n=10)
Cutoff (131.48)
Raw p=0.25
c
CXCR2
High (n=50)
Low (n=50)
Cutoff (25.83)
Raw p=0.13
High (n=29)
Low (n=33)
Cutoff (23.77)
Raw p=0.15
High (n=30)
Low (n=8)
Cutoff (7.79)
Raw p=0.21
High (n=12)
Low (n=22)
Cutoff (30.66)
Raw p=0.15
Months

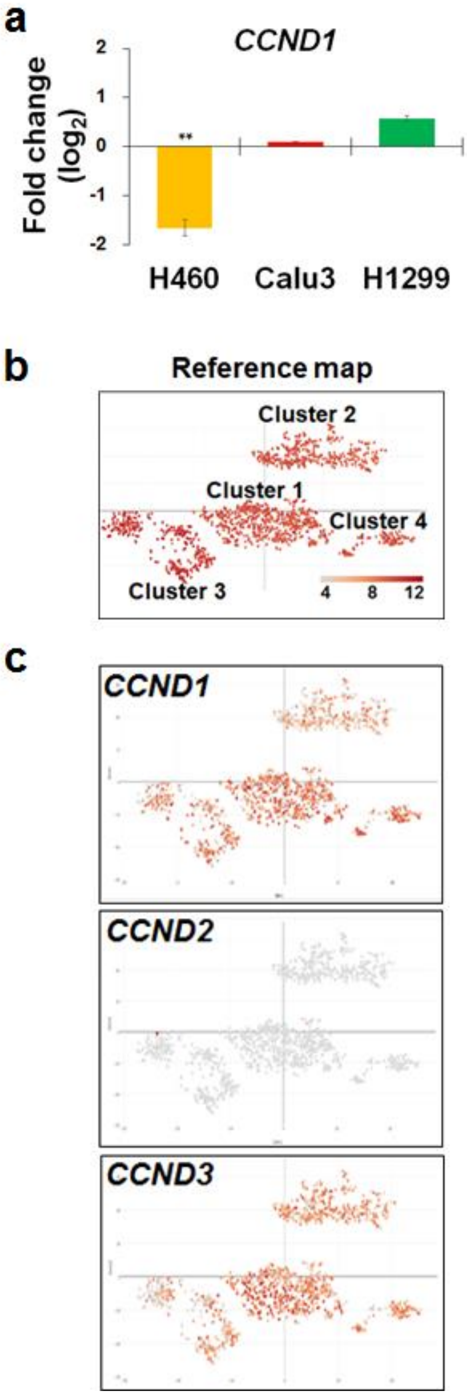
a
CCND1
Fold change (log2)
2
1
0
-1
-2
**
H460
Calu3
H1299
b
Reference map
Cluster 2
Cluster 1
Cluster 4
Cluster 3
4
8
12
c
CCND1
CCND2
CCND3

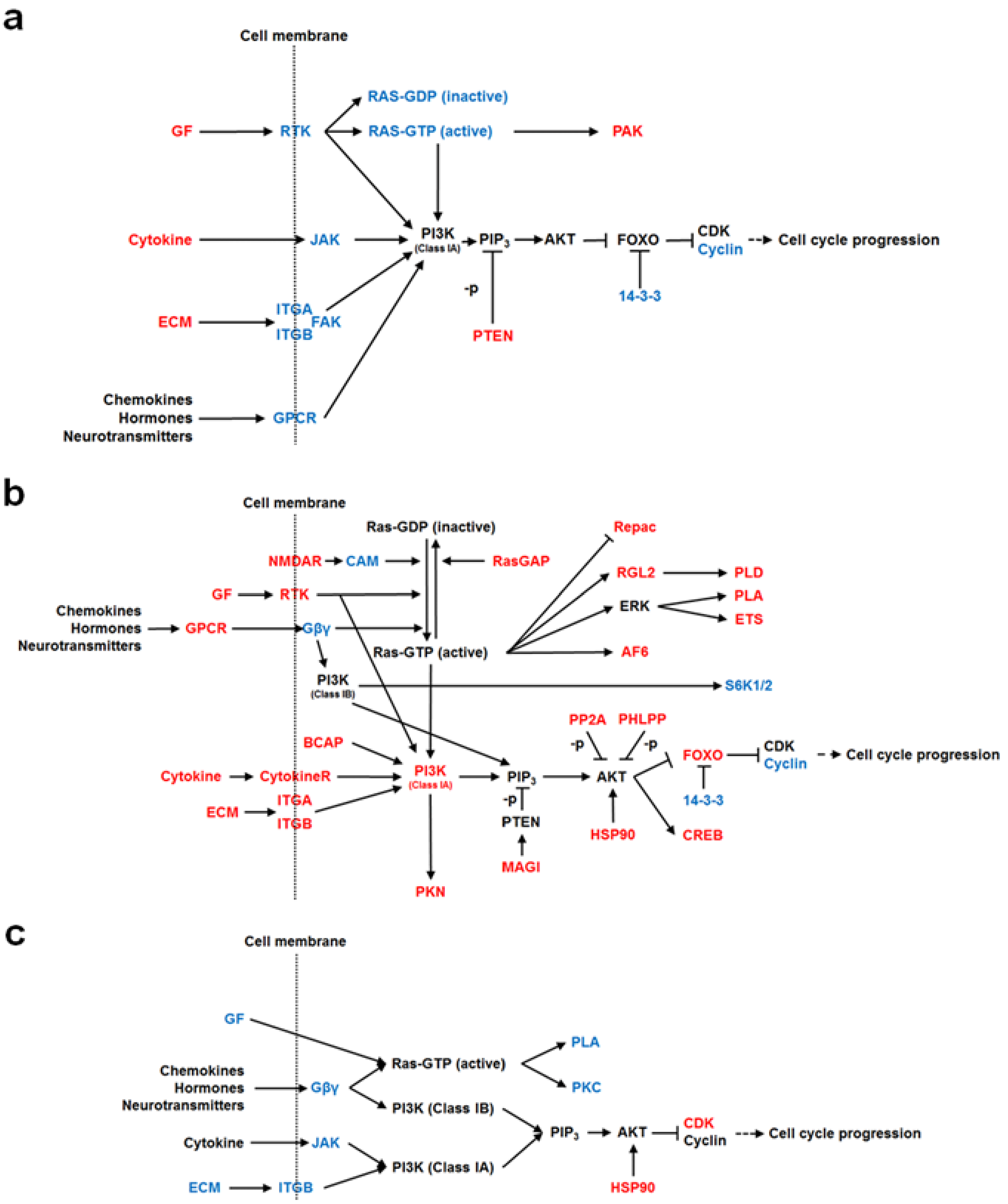
a
Cell membrane
RAS-GDP (inactive)
GF
RTK
RAS-GTP (active)
PAK
Cytokine
JAK
PI3K
(Class IA)
PIP_3
AKT
FOXO
CDK
Cyclin
Cell cycle progression
-p
14-3-3
ECM
ITGA
ITGB
FAK
PTEN
Chemokines
Hormones
Neurotransmitters
GPCR
b
Cell membrane
Ras-GDP (inactive)
Repac
NMDAR
CAM
RasGAP
RGL2
PLD
GF
RTK
ERK
PLA
ETS
Chemokines
Hormones
Neurotransmitters
GPCR
Gβγ
Ras-GTP (active)
AF6
PI3K
(Class IB)
S6K1/2
PP2A
PHLPP
-p
-p
BCAP
FOXO
CDK
Cyclin
Cell cycle progression
Cytokine
CytokineR
PI3K
(Class IA)
PIP_3
AKT
14-3-3
ECM
ITGA
ITGB
-p
PTEN
HSP90
CREB
MAGI
PKN
c
Cell membrane
GF
PLA
Ras-GTP (active)
Chemokines
Hormones
Neurotransmitters
Gβγ
PKC
PI3K (Class IB)
PIP_3
AKT
CDK
Cyclin
Cell cycle progression
Cytokine
JAK
PI3K (Class IA)
ECM
ITGB
HSP90

Supplementary Figure Legends

Supplementary Figure 1: Human genes mapped by Fluidigm 3'-end scRNA-seq dataset from four NSCLC cell lines. a The average number of genes mapped with sequence reads per cell line. Lines and x marks in individual boxes indicate mean and median values, respectively. Data is expressed mean ± SEM. **b** The number of mapped genes per single cell after normalizing read counts per gene at counts per million reads (CPM) ≥ 1.

Supplementary Figure 2: Measured quantities of *CXCL1*, *CXCL2* and *CXCR2* in primary lung tumors and normal lung tissues resected from Stage I LUAD patients. a-c Absolute quantification of *CXCL1* in **a**, *CXCL2* in **b** and *CXCR2* in **c** from normal (black bars) and tumor (grey bars) tissues of female (*n* = 16) and male (*n* = 18) Stage I LUAD patients. X and y axes present individual patient IDs (female patients, 1-16, and male patients, 17-34) and measured quantities (ng), respectively. Data is expressed mean ± SEM from three separate experiments conducted in duplicate. FS2 supports Fig. 5.

Supplementary Figure 3: Measured quantities of microRNAs in primary lung tumors and adjacent normal lung tissues resected from Stage I LUAD patients. a-c Absolute quantification of miR-532-5p in **a**, miR-1266-3p in **b** and miR-3163 in **c** from normal (black bars) and tumor (grey bars) tissues of female (*n* = 16) and male (*n* = 18) Stage I LUAD patients. X and y axes present individual patient IDs (female patients, 1-16, and male patients, 17-34) and measured quantities (ng), respectively. Data is expressed mean ± SEM from three separate experiments conducted in duplicate. FS3 supports Fig. 5.

Supplementary Figure 4: Reference expression map mined from publicly available LUAD 10x Genomics 3'-end scRNA-seq dataset. Cluster plot of single cells (*n* = 208,506) from multiple-stage LUAD patients (*n* = 58). Clusters were generated from 10x Genomics 3'-end scRNA-seq dataset (GSE131907)[16]. FS4 supports Fig. 7.

Supplementary Figure 5: Survival analysis of chemokine genes in primary tumors from LUAD patients. a-c Kaplan-Meier survival plots for differential expression of *CXCL1* in **a**, *CXCL2* in **b** and *CXCR2* in **c** obtained from mined public bulk RNA-seq datasets of primary tumor tissues resected from female LUAD patients (early-Stage I; *n* = 100) and (late-Stage III & IV; *n* = 38), and male LUAD patients (early-Stage I; *n* = 62) and (late-Stage III & IV; *n* = 34). X and y axes indicate follow up in months and survival probability, respectively. The cutoff expression value between high and low expression per sex, stage and gene was determined by a log-rank test. FS5 supports Fig. 7.

Supplementary Figure 6: Validation of cyclin genes from human NSCLC epithelial cell lines. a Fold change validation of cyclin D1 (*CCND1*) in H460 (orange), Calu3 (red) and H1299 (green) compared with A549 (control cell line) and *ACTG1* (endogenous control gene) using a qRT-PCR analysis. Fold change values presented as mean ± SEM from three separate experiments conducted in duplicate. **P < 0.01 from two-sample *t*-tests for measured quantities. **b** Reference clusters map generated from single cells (*n* = 1,441) from the four NSCLC cell lines. **c** Expression maps for *CCND1*, *CCND2* and *CCND3*.

Supplementary Figure 7: RAS-PI3K-AKT signaling pathways for cell cycle-associated DEGs. a-c RAS-PI3K-AKT signaling pathway encoded by up (n = 10) and down (n = 12)-regulated genes in Cluster 2 in **a**, up-regulated (n = 44) and down-regulated (n = 10) genes in Cluster 3 in **b**, and up-regulated (n = 3) and down-regulated (n = 8) genes in Cluster 4 in **c** compared with Cluster 1. Up- and down-regulated protein expression (Red and blue, respectively).

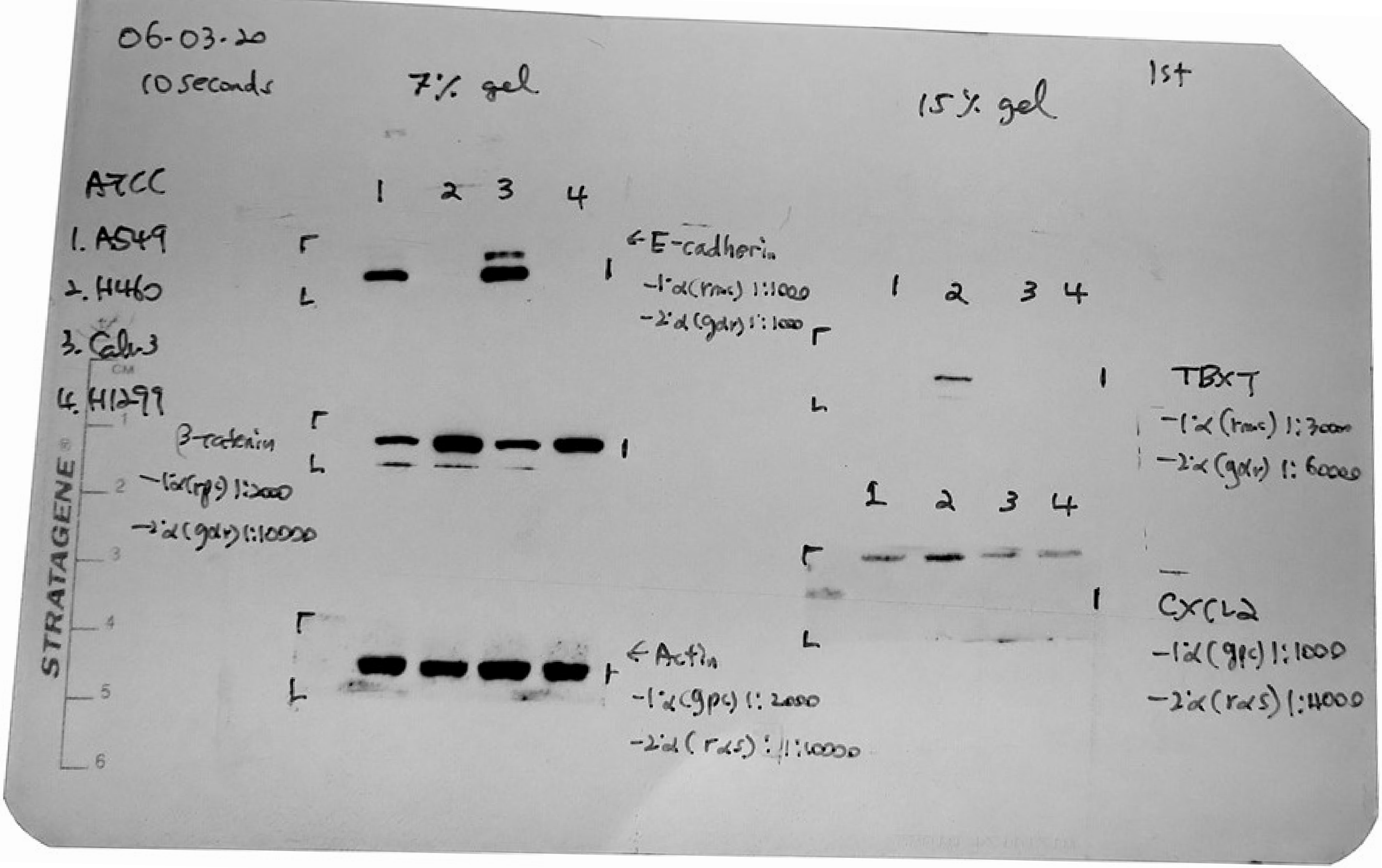
06-03-20
10 seconds
7% gel
1st
15% gel
ATCC
1. A549
2. H460
3. Calu3
4. H1299
1 2 3 4
← E-cadherin
-1°α(rmc) 1:1000
-2°α(gαr) 1:1000
1 2 3 4
TBXT
-1°α(rmc) 1:3000
-2°α(gαr) 1:6000
β-catenin
-1°α(rpg) 1:2000
-2°α(gαr) 1:10000
1 2 3 4
CXCL2
-1°α(gpc) 1:1000
-2°α(rαs) 1:4000
← Actin
-1°α(gpc) 1:2000
-2°α(rαs) 1:10000
STRATAGENE
CM
1
2
3
4
5
6
