# Supplementary material for: Single-cell RNA sequencing for the identification of early-stage lung cancer biomarkers from circulating blood
Source: NPJ Genom Med. 2021 Oct 15;6:87. doi: 10.1038/s41525-021-00248-y (PMC8519939; doi:10.1038/s41525-021-00248-y)
Supplement: Supplementary file 8 — Reporting Summary [file 41525_2021_248_MOESM8_ESM.pdf]

nature portfolio

Corresponding author(s): Marignani, Paola A.

Last updated by author(s): Jul 29, 2021

Reporting Summary

Nature Portfolio wishes to improve the reproducibility of the work that we publish. This form provides structure for consistency and transparency in reporting. For further information on Nature Portfolio policies, see our Editorial Policies and the Editorial Policy Checklist.

Statistics

For all statistical analyses, confirm that the following items are present in the figure legend, table legend, main text, or Methods section.

n/a	Confirmed	
☐	☒	The exact sample size (*n*) for each experimental group/condition, given as a discrete number and unit of measurement
☐	☒	A statement on whether measurements were taken from distinct samples or whether the same sample was measured repeatedly
☐	☒	The statistical test(s) used AND whether they are one- or two-sided<br>*Only common tests should be described solely by name; describe more complex techniques in the Methods section.*
☒	☐	A description of all covariates tested
☒	☐	A description of any assumptions or corrections, such as tests of normality and adjustment for multiple comparisons
☐	☒	A full description of the statistical parameters including central tendency (e.g. means) or other basic estimates (e.g. regression coefficient) AND variation (e.g. standard deviation) or associated estimates of uncertainty (e.g. confidence intervals)
☐	☒	For null hypothesis testing, the test statistic (e.g. *F*, *t*, *r*) with confidence intervals, effect sizes, degrees of freedom and *P* value noted<br>*Give P values as exact values whenever suitable.*
☒	☐	For Bayesian analysis, information on the choice of priors and Markov chain Monte Carlo settings
☒	☐	For hierarchical and complex designs, identification of the appropriate level for tests and full reporting of outcomes
☒	☐	Estimates of effect sizes (e.g. Cohen's *d*, Pearson's *r*), indicating how they were calculated

Our web collection on statistics for biologists contains articles on many of the points above.

Software and code

Policy information about availability of computer code

Data collection	1. C1 running script: mRNA Seq HT:RT & Amp (1912x) (Fluidigm, Inc.).<br>2. Bulk RNA-seq data collection: Cancer Genome Atlas (TCGA) program (RRID:SCR_003193).<br>3. Bulk RNA-seq data collection: Genomic Data Commons Data Portal (RRID:SCR_014514).

Data analysis

1. Demultiplexing tool: C1 mRNA Sequencing High Throughput Demultiplexer Script (Fluidigm, Inc.).
2. Demultiplexing tool: Geneious Prime 2019.2.1 (https://www.geneious.com).
3. Raw sequence read processing tool: BBDuk Trimmer (a part of Bestus Bioinformatics Tools; RRID:SCR_016968).
4. Read mapping tool: HISAT2 (RRID:SCR_015530).
5. Mapped reads-counting tool: FeatureCounts (A part of Subread; RRID:SCR_009803).
6. Single-cell data analysis tool: ASAP (https://asap.epfl.ch/).
7. Read-scaling tool: voom (Law et al., 2014; https://genomebiology.biomedcentral.com/articles/10.1186/gb-2014-15-2-r29) equipped to ASAP.
8. Clustering tool: SC3 (RRID:SCR_015953) equipped to ASAP.
9. Heatmap-clustering tool: Morpheus by Broad Institute (RRID:SCR_017386).
10. Volcano plot presentation tool: GraphPad Prism (RRID:SCR_002798).
11. Gene set enrichment analysis database and tool for gene ontology: PANTHER (RRID:SCR_004869).
12. Gene set enrichment analysis database for signaling pathways: KEGG (RRID:SCR_012773).
13. Gene set enrichment analysis database for oncogenic gene sets: Molecular Signatures (RRID:SCR_016863).
14. Primer collection database: PrimerBank (RRID:SCR_006898).
15. Primer collection database: RTPrimerDB-The Real-Time PCR and Probe Database (RRID:SCR_007106).
16. Primer-designing tool: Primer3 (RRID:SCR_003139).
17. microRNA database: miRDB (RRID:SCR_010848).
18. Survival plot generation tool: R2 genomics analysis and visualization platform (https://hgserver1.amc.nl/cgi-bin/r2/main.cgi).

For manuscripts utilizing custom algorithms or software that are central to the research but not yet described in published literature, software must be made available to editors and reviewers. We strongly encourage code deposition in a community repository (e.g. GitHub). See the Nature Portfolio guidelines for submitting code & software for further information.

Data

Policy information about availability of data

All manuscripts must include a data availability statement. This statement should provide the following information, where applicable:

- Accession codes, unique identifiers, or web links for publicly available datasets
- A description of any restrictions on data availability
- For clinical datasets or third party data, please ensure that the statement adheres to our policy

Demultiplexed single-cell RNA-seq dataset generated from the current study was deposited to Gene Expression Omnibus (NCBI); GSE183590

Field-specific reporting

Please select the one below that is the best fit for your research. If you are not sure, read the appropriate sections before making your selection.

☒ Life sciences ☐ Behavioural & social sciences ☐ Ecological, evolutionary & environmental sciences

For a reference copy of the document with all sections, see nature.com/documents/nr-reporting-summary-flat.pdf

Life sciences study design

All studies must disclose on these points even when the disclosure is negative.

Sample size

1. Human lung adenocarcinoma (LUAD) cell lines: We chose four human LUAD cell lines based on mutation on KRAS and TP53 genes which are frequently found in most LUAD cases. Both A549 and H460 cell lines have mutated KRAS and wild type TP53, while H1299 and Calu3 express wild-type KRAS and mutated TP53.

2. Human LUAD patient tissues and blood samples: We validated fold differences of three chemokine genes in primary tumor and tumor-adjacent normal lung tissues of female (n = 16) and male (n = 18) early-stage (Stage I) LUAD patients. We also validated miRNA expression in those tissues as well as blood (plasma) samples from corresponding patients.

In brief, we used the four LUAD cell lines to conduct scRNA-seq, then selected three chemokine genes of interest. Following this we expanded sample size to the 34 early-stage LUAD patients at validation process.

Data exclusions

No data were excluded.

Replication

As described in the manuscript, we used two replicates per cDNA sample and repeated three times per gene, including standard curves, for qRT-PCR analyses to validate fold difference of selected genes. We presented dataset only when PCR amplicons per gene were consistently detectable for the repeats.

Randomization

We did not allocate cell lines and patient samples following randomization test as the cell lines were chosen according to absence and presence of mutations in KRAS and TP53 genes and the patient samples were retrieved from surgical suite whenever patient underwent surgical resection for their lung cancer.

Blinding

The only information we were provided at the time of surgical resection was that the patient had lung cancer.

Reporting for specific materials, systems and methods

We require information from authors about some types of materials, experimental systems and methods used in many studies. Here, indicate whether each material, system or method listed is relevant to your study. If you are not sure if a list item applies to your research, read the appropriate section before selecting a response.

Materials & experimental systems

n/a	Involved in the study
☐	☒ Antibodies
☐	☒ Eukaryotic cell lines
☒	☐ Palaeontology and archaeology
☒	☐ Animals and other organisms
☐	☒ Human research participants
☒	☐ Clinical data
☒	☐ Dual use research of concern

Methods

n/a	Involved in the study
☒	☐ ChIP-seq
☒	☐ Flow cytometry
☒	☐ MRI-based neuroimaging

Antibodies

Antibodies used

1. CXCL2 (Cat. No. ab91511, Abcam).
2. Brachyury (Cat. No. 81694, Cell Signaling Technology).
3. E-cadherin (Cat. No. 3195, Cell Signaling Technology).
4. β-catenin (Cat. No. 9562, Cell Signaling Technology).
5. actin (Cat. No. ab8229, Abcam).

Validation

1. CXCL2: We used the primary antibody to validate its gene expression at protein level using western blotting (WB) analysis. Host species was goats (polyclonal), and it was recommended to use for WB analysis with human protein. Manufacturer (abcam, Inc.) validated CXCL2 expression at 11~17 kDa in Human duodenum lysate using WB analysis with the antibody. Please refer to more detailed information, including references, in the following link (https://www.abcam.com/cxcl2-antibody-ab91511.html).

2. Brachyury: We used the primary antibody to validate its gene (TBXT) expression at protein level using WB analysis. Host species was rabbits (monoclonal), and it was recommended to use for analyses of WB, immunoprecipitation (IP), immunofluorescence (IF) and flow cytometry (F) with human protein. Manufacturer (Cell Signaling Technology, Inc.) validated Brachyury expression at 54 kDa in NCI-H460 (human), MUG-CHOR-1 (human) and UCH2 (human) cell lines using WB analysis with the antibody. Please refer to more detailed information, including references, in the following link (https://www.cellsignal.com/products/primary-antibodies/brachyury-d2z3j-rabbit-mab/81694).

3. E-cadherin: We used the primary antibody to validate its gene (CDH1) expression at protein level using WB analysis. Host species was rabbits (monoclonal), and it was recommended to use for analyses of WB, immunohistochemistry (IHC), IP, IF and F with human and mouse proteins. Manufacturer (Cell Signaling Technology, Inc.) validated Brachyury expression at 135 kDa in MCF7 (human), HPAC (human), mIMCD3 (mouse) and CSC12 (mouse) cell lines using WB analysis with the antibody. Please refer to more detailed information, including references, in the following link (https://www.cellsignal.com/products/primary-antibodies/e-cadherin-24e10-rabbit-mab/3195).

4. β-catenin: We used the primary antibody to validate its gene (CTNNB1) expression at protein level using WB analysis. Host species was rabbits (polyclonal), and it was recommended to use for analyses of WB, IHC and IP with human, mouse, rat and monkey proteins. Manufacturer (Cell Signaling Technology, Inc.) validated β-catenin expression at 92 kDa in HEK293 (human), HeLa (human), NIH/3T3 (mouse), C6 (rat) and SW480 (human) cell lines using WB analysis with the antibody. Please refer to more detailed information, including references, in the following link (https://www.cellsignal.com/products/primary-antibodies/b-catenin-antibody/9562).

5. Actin: We used the primary antibody as a loading control using WB analysis. Host species was goats (polyclonal), and it was recommended to use for WB analysis with human, mouse, rat, rabbit, cow, dog and Chinese hamster proteins. Manufacturer (abcam, Inc.) validated actin expression at 42 kDa in HeLa whole cell (human), 3T3 cell (mouse), Rabbit Liver, MDCK cell (dog), EBTr cell (cow), SL-29 cell (chicken), CHO cell (Chinese Hamster) using WB analysis with the antibody. Please refer to more detailed information, including references, in the following link (https://www.abcam.com/beta-actin-antibody-loading-control-ab8229.html).

Eukaryotic cell lines

Policy information about cell lines

Cell line source(s)

We purchased the four human NSCLC epithelial cell lines, A549 (ATCC-CCL-185), H460 (ATCC-HTB-177), Calu3 (ATCC-HTB-55) and H1299 (ATCC-CRL-5803) from ATCC (VA, USA).

Authentication

The cell lines were used directly from ATCC. No expansion or culturing involved.

Mycoplasma contamination

The cells are guaranteed to be mycoplasma free when purchased from ATCC. We processed the cells immediately for scRNAseq.

Commonly misidentified lines (See ICLAC register)	*Name any commonly misidentified cell lines used in the study and provide a rationale for their use.*

Human research participants

Policy information about studies involving human research participants

Population characteristics	Please find the information in the Supplementary Table 8 containing the sex, mutation and smoking status of blood- and tissue-donor patients.
Recruitment	Blood- and tissue-donor patients voluntarily signed consent when asked that their tumor and blood be donation for the purpose of research.
Ethics oversight	Nova Scotia Health Authority (REB # 1024460)

Note that full information on the approval of the study protocol must also be provided in the manuscript.
